# Effect of sorafenib maintenance on Epstein-Barr virus and cytomegalovirus infections in patients with FLT3-ITD AML undergoing allogeneic hematopoietic stem cell transplantation: a secondary analysis of a randomized clinical trial

**DOI:** 10.1186/s12916-022-02479-x

**Published:** 2022-09-02

**Authors:** Xin Xu, Zhiping Fan, Yu Wang, Fen Huang, Yajing Xu, Jing Sun, Na Xu, Lan Deng, Xudong Li, Xinquan Liang, Xiaodan Luo, Pengcheng Shi, Hui Liu, Yan Chen, Sanfang Tu, Xiaojun Huang, Qifa Liu, Li Xuan

**Affiliations:** 1 Department of Hematology, Nanfang Hospital, Southern Medical University, Guangzhou, 510515 China; 2 Institute of Hematology, Peking University People’s Hospital, Beijing, 100044 China; 3 Department of Hematology, Xiangya Hospital, Central South University, Changsha, China; 4 Department of Hematology, Zhujiang Hospital, Southern Medical University, Guangzhou, China; 5 Department of Hematology, the Third Affiliated Hospital of Sun Yat-Sen University, Guangzhou, China; 6 Department of Hematology, the First People’s Hospital of Chenzhou, Chenzhou, China; 7 Department of Hematology, the First Affiliated Hospital of Guangzhou Medical University, Guangzhou, China

**Keywords:** Sorafenib, Epstein-Barr virus, Cytomegalovirus, FLT3-ITD acute myeloid leukemia, Allogeneic hematopoietic stem cell transplantation

## Abstract

**Background:**

Use of kinase inhibitors such as dasatinib and imatinib might increase the risk of opportunistic infections, especially Epstein-Barr virus (EBV) and cytomegalovirus (CMV) infections. However, the effect of sorafenib on EBV and CMV infections remains unclear. The aim of this study was to investigate the effect of sorafenib maintenance post-transplantation on the incidence and mortality of EBV and CMV infections in patients with FLT3-ITD acute myeloid leukemia.

**Methods:**

This was a follow-up of our randomized controlled trial undertaken at seven hospitals in China. The primary endpoint was EBV and CMV infections within 3 years post-transplantation. Secondary endpoints included the cumulative incidences of relapse, non-relapse mortality (NRM), overall survival (OS), leukemia-free survival (LFS), and graft-versus-host disease (GVHD)-free/relapse-free survival (GRFS) at 3 years.

**Results:**

Two hundred two patients were assigned to sorafenib maintenance (*n*=100) or non-maintenance (control, *n*=102). Median extended follow-up post-transplantation was 36.8 (range, 2.5–67.1) months. The 3-year cumulative incidences of EBV-DNAemia and EBV-associated diseases were 24.0% (95% CI: 16.1–32.8%) and 5.0% (1.8–10.6%) in the sorafenib group, and 24.5% (16.6–33.2%) and 5.9% (2.4–11.6%) in the control group (*P*=0.937; *P*=0.771). The 3-year cumulative incidences of CMV-DNAemia and CMV-associated diseases were 56.0% (45.6–65.1%) and 8.0% (3.7–14.4%) in the sorafenib group, and 52.9% (42.7–62.1%) and 8.8% (4.3–15.3%) in the control group (*P*=0.997; *P*=0.826). The 3-year cumulative mortality of EBV- and CMV-associated diseases was 0.0% (0.0–0.0%) and 2.0% (0.4–6.4%) in the sorafenib group, and 1.0% (0.1–4.8%) and 2.0% (0.4–6.3%) in the control group (*P*=0.322, *P*=0.980). The 3-year cumulative incidences of relapse, NRM, OS, LFS, and GRFS were 13.0%, 11.1%, 79.0%, 75.9%, and 65.8% in the sorafenib group and 34.8%, 12.7%, 61.4%, 52.5%, and 46.6% in the control group, respectively (*P*<0.001, *P*=0.656, *P*=0.005, *P*<0.001, *P*=0.003). The reconstitution of T lymphocyte subsets, B lymphocytes, and natural killer cells was similar between the two groups (all *P*>0.05).

**Conclusions:**

Sorafenib maintenance post-transplantation does not increase the incidence and mortality of EBV and CMV infections, demonstrating a favorable safety profile.

**Trial registration:**

ClinicalTrials.gov Identifier: NCT02474290. Registered on June 14, 2015

**Supplementary Information:**

The online version contains supplementary material available at 10.1186/s12916-022-02479-x.

## Background

FLT3 internal tandem duplication (FLT3-ITD) mutations occur in approximately 25% of adult acute myeloid leukemia (AML) patients and usually confer an adverse prognosis [1]. A growing body of research has shown that allogeneic hematopoietic stem cell transplantation (allo-HSCT) could improve the survival for these patients [2, 3]. Despite this, the relapse rate remains high [2, 3]. Several retrospective and prospective studies including our randomized controlled trial (RCT) have demonstrated that sorafenib maintenance post-transplantation can prevent relapse and improve survival for patients with FLT3-ITD AML undergoing allo-HSCT [4–10].

Sorafenib, a multi-kinase inhibitor, blocks multiple pathways involved in the development and progression of AML, such as FLT3-ITD, Ras/Raf, c-KIT as well as vascular endothelial growth factor and platelet-derived growth factor receptors (PDGFR) [3]. Recently, multiple studies have revealed that use of kinase inhibitors such as dasatinib and imatinib might affect the immune function and increase the risk of opportunistic infections, especially Epstein-Barr virus (EBV) and cytomegalovirus (CMV) infections [11–20]. Some studies reported that sorafenib could inhibit CMV replication and induce apoptosis in EBV-transformed B cells in vitro [21, 22]. However, the effect of sorafenib on EBV and CMV infections in sorafenib-treated patients remains unclear. In order to clarify the issue, we analyzed the prospective RCT to explore the incidence and mortality of EBV and CMV infections in patients with FLT3-ITD AML with and without sorafenib maintenance post-transplantation.

## Methods

### Study design and participants

**Fig. 1:**
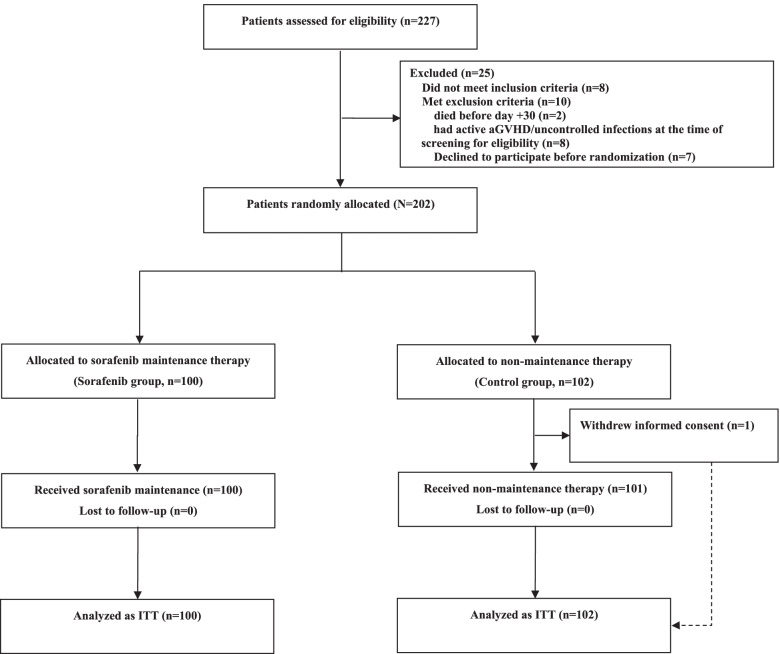
CONSORT flow diagram. aGVHD, acute graft-versus-host disease; ITT, intention to treat

An open-label, randomized phase 3 trial was conducted at seven hospitals in China to investigate the efficacy and tolerability of sorafenib maintenance post-transplantation for prevention of relapse in patients with FLT3-ITD AML undergoing allo-HSCT. The trial design was reported in detail previously [6], and the study protocol is shown in Additional file 1: Study protocol. The trial was registered at clinicaltrials.gov (NCT02474290). Briefly, patients with FLT3-ITD AML undergoing first allo-HSCT who were aged 18–60 years, had an Eastern Cooperative Oncology Group performance status of 0–2, had composite complete remission before and after allo-HSCT, and had hematopoietic recovery within 60 days post-transplantation were eligible for inclusion in the trial. After enrolment, patients were randomized 1:1 to sorafenib maintenance or non-maintenance at 30–60 days post-transplantation (Fig. 1). For patients assigned to sorafenib maintenance (sorafenib group), sorafenib was administered at 30–60 days post-transplantation and continued until day 180. For patients assigned to non-maintenance (control group), neither sorafenib nor other FLT3 inhibitors were used, unless the patient experienced relapse. The study protocol was in accordance with the Declaration of Helsinki and was approved by the local ethics committee review board. Written informed consent was obtained from the participants in accordance with the modified Helsinki Declaration. This was a long-term follow-up study of the RCT, and the primary objective was to explore the effect of sorafenib maintenance on EBV and CMV infections in patients with FLT3-ITD AML post-transplantation.

### Procedures

All patients received myeloablative conditioning with a modified busulfan-cyclophosphamide regimen [6]. Patients with haploidentical donor (HID) were transplanted with a combination of bone marrow (BM) and peripheral blood stem cell (PBSC) grafts, and patients with HLA-matched sibling donor (MSD) or HLA-matched unrelated donor (MUD) received PBSC grafts. Cyclosporine A (CsA), methotrexate (MTX), and mycophenolate (MMF) were administered to patients undergoing MSD transplants for graft-versus-host disease (GVHD) prophylaxis. CsA, MTX, and antithymocyte immunoglobulin (ATG) were administered to patients undergoing MUD transplants. CsA, MTX, ATG, and MMF were administered to patients undergoing HID transplants [6]. The dose of rabbit ATG (Thymoglobulin, Imtix Sangstat, Lyon, France) was 7.0mg/kg in MUD transplants and 7.5mg/kg in HID transplants.

Infection prophylaxis was administered as previously described [23]. Oral sulfamethoxazole and norfloxacin were given to all patients. Oral fluconazole was used for up to +60 days post-transplantation in patients with no history of invasive fungal infection (IFI); for patients with a history of IFI, antifungal agents for secondary prophylaxis based on response to initial antifungal therapy were used for up to +90 days post-transplantation. Acyclovir was given daily from the beginning of conditioning to engraftment and then administered daily for 7 days every 2 weeks until 1 year post-transplantation.

The EBV- and CMV-DNA loads in the peripheral blood (PB) were measured by quantitative real-time polymerase chain reaction (PCR) weekly for the first 3 months, once every 2 weeks from 4th to 9th month, once every month from 10th to 12th month, and once every 3 months from 13th to 36th after allo-HSCT. If EBV/CMV-DNA in PB was positive, it was monitored twice a week. The method of PCR detection has been reported previously [24, 25]. The threshold for EBV/CMV-DNA copies in plasma provided by the manufacturer (ZJ Bio-Tech Co., Ltd., Shanghai, China) was less than 500 copies/ml.

Pre-emptive therapy of EBV- and CMV-DNAemia was conducted according to our previous report [25]. Immunosuppressants were reduced when the condition of the patients was permitted. Rituximab was used as preemptive therapy of EBV-DNAemia, and ganciclovir or foscarnet was used as preemptive therapy of CMV-DNAemia. For the patients who had non-complete response after 4 weeks of preemptive therapy, adoptive cellular immunotherapies, including EBV/CMV-specific cytotoxic T lymphocyte (CTL) or donor lymphocyte infusion (DLI), were used.

The treatment of EBV/CMV-associated diseases was based on our previous report [24]. When EBV-associated diseases were diagnosed, rituximab-based treatments would be taken promptly, including reduction of immunosuppressants, rituximab, chemotherapy, EBV-CTL, or DLI. Once CMV-associated diseases were diagnosed, several measures would be taken promptly, including administration of antiviral agents such as ganciclovir or foscarnet, immunoglobulin or hyperimmune globulin to antiviral therapy, reduction of immunosuppressants, or CMV-CTL.

BM assessments were done before randomization, every month for the first 3 months after enrolment, every 2 months from month 4 to month 9 after enrolment, and then every 3 months until the study was completed. Eight-color multi-parameter flow cytometry and quantitative PCR were used for detection of minimal residual disease. Immune reconstitution was assessed as previously described [24]. Briefly, T lymphocyte subsets (CD3^+^, CD3^+^CD4^+^, CD3^+^CD8^+^), B lymphocytes (CD19^+^), and natural killer (NK) cells (CD3^-^CD56^+^) in the PB of recipients were analyzed by flow cytometry, respectively, at 1, 3, 6, 9, and 12 months after allo-HSCT.

### Outcomes

The primary endpoint was EBV and CMV infections within 3 years post-transplantation, including EBV and CMV-DNAemia and diseases. EBV- or CMV-DNAemia was defined as EBV- or CMV-DNA in PB positive twice consecutively. The diagnosis of EBV- and CMV-associated diseases was based on the guidelines for the management of EBV and CMV infections [26, 27]. Secondary endpoints included the cumulative incidences of relapse, non-relapse mortality (NRM), overall survival (OS), leukemia-free survival (LFS), and GVHD-free/relapse-free survival (GRFS) at 3 years. Relapse, NRM, OS, and LFS were assessed as previously described [6]. GRFS was defined as time from transplantation until grade III–IV acute GVHD (aGVHD), chronic GVHD (cGVHD) requiring systemic immunosuppressive therapy, leukemia relapse, or death from any cause [28].

### Statistical analysis

Our data were analyzed on December 31, 2020. Categorical variables were compared using the *χ*^2^ test, and continuous variables were compared using the Mann-Whitney *U* test. Cumulative incidences of relapse, NRM, and EBV/CMV infections were calculated with the Fine-Gray competing risk method [29]. Competing events were defined as follows: for NRM, relapse; for relapse, NRM; for EBV-DNAemia/associated diseases, death without EBV-DNAemia/associated diseases and relapse requiring further therapy; and for CMV-DNAemia/ associated diseases, death without CMV-DNAemia/associated diseases and relapse requiring further therapy. OS, LFS, and GRFS were estimated using the Kaplan-Meier method and compared using the log-rank test. The corresponding hazard ratio (HR) and 95% CI were estimated using the Cox proportional hazards model. The Cox proportional hazards model was also used for the analysis of risk factors for EBV and CMV infections. In these regression models, the occurrence of acute and chronic GVHD was treated as a time-dependent covariate. In the analysis of aGVHD, patients were assigned to the “no aGVHD group” at the time of transplantation and then transferred to the “aGVHD group” at the onset of aGVHD, without considering the occurrence of cGVHD. The analysis of cGVHD included patients who survived at least 100 days without relapse. Patients were assigned to the “no cGVHD group” at the time of transplantation and then transferred to the “cGVHD group” at the onset of cGVHD. Other variables included in the univariable analysis were gender, age, EBV and CMV serostatus, complete remission (CR) status at transplantation, use of ATG in the conditioning, and sorafenib use pre-transplantation and post-transplantation. Only variables with a *P* value less than 0.10 were included in the multivariable analysis. All *P* values were two-sided with a significance level of 0.05. SPSS 20.0 and R version 3.3.0 were used for data analysis.

## Results

### Study population

A total of 202 patients with FLT3-ITD AML (sorafenib, *n*=100; control, *n*=102) were recruited from June 20, 2015, to July 21, 2018. The median age was 35 (range: 18–60) years, with 102 males and 100 females. Patient characteristics are summarized in Table 1. Baseline factors were well balanced between the two groups. With a median of 18 days after sorafenib initiation, 59 of 100 patients required dose modifications due to adverse events, including 42 dose reductions, 12 dose interruptions, and 5 discontinuations. The median follow-up was 36.8 (range, 2.5–67.1) months post-transplantation.

**Table 1:** Patient and transplant characteristics

Patient characteristics	Sorafenib group (***n***=100)	Control group (***n***=102)	***P*** value
**Gender, male/female**	**50 (50.0%)/50 (50.0%)**	**52 (51.0%)/50 (49.0%)**	**0.889**
**Patient age, median (range), years**	**35 (18–60)**	**35 (18–59)**	**0.916**
**WBC at diagnosis, median (range)**	**54.8 (1.3–463.0)**	**74.8 (1.7–385.1)**	**0.473**
**Cytogenetic risk**^#^			**0.818**
**Low risk**	**6 (6.0%)**	**4 (3.9%)**	
**Intermediate risk**	**80 (80.0%)**	**85 (83.3%)**	
**High risk**	**7 (7.0%)**	**5 (4.9%)**	
**Unknown**	**7 (7.0%)**	**8 (7.8%)**	
**NPM1 mutation**			**0.575**
**Concomitant**	**29 (29.0%)**	**26 (25.5%)**	
**Without**	**71 (71.0%)**	**76 (74.5%)**	
**EBV serostatus**			**0.910**
**D−/R-**	**21 (21.0%)**	**19 (18.6%)**	
**D+/R-**	**24 (24.0%)**	**28 (27.5%)**	
**D−/R+**	**20 (20.0%)**	**22 (21.6%)**	
**D+/R+**	**35 (35.0%)**	**33 (32.3%)**	
**CMV serostatus**			**0.982**
**D−/R−**	**7 (7.0%)**	**7 (6.9%)**	
**D+/R−**	**5 (5.0%)**	**4 (3.9%)**	
**D−R+**	**9 (9.0%)**	**10 (9.8%)**	
**D+/R+**	**79 (79.0%)**	**81 (79.4%)**	
**Sorafenib pre-transplant**			**0.654**
**Use**	**59 (59.0%)**	**57 (55.9%)**	
**No use**	**41 (41.0%)**	**45 (44.1%)**	
**CR status at transplant**			**0.546**
**≥ CR2**	**21 (21.0%)**	**18 (17.6%)**	
**CR1**	**79 (79.0%)**	**84 (82.4%)**	
**MRD at transplant**			**0.723**
**Positive**	**31 (31.0%)**	**34 (33.3%)**	
**Negative**	**69 (69.0%)**	**68 (66.7%)**	
**GVHD prophylaxis**			**0.512**
**CsA+MTX+MMF**	**44 (44.0%)**	**39 (38.2%)**	
**CsA+MTX+ATG**	**8 (8.0%)**	**6 (5.9%)**	
**CsA+MTX+ATG+MMF**	**48 (48.0%)**	**57 (55.9%)**	
**ATG use in the conditioning**			**0.405**
**Use**	**44 (44.0%)**	**39 (38.2%)**	
**No use**	**56 (56.0%)**	**63 (61.8%)**	
**Transplant modality**			**0.512**
**MSD**	**44 (44.0%)**	**39 (38.2%)**	
**MUD**	**8 (8.0%)**	**6 (5.9%)**	
**HID**	**48 (48.0%)**	**57 (55.9%)**	
**Donor sources**			**0.262**
**PBSC**	**52 (52.0%)**	**45 (44.1%)**	
**PBSC+BM**	**48 (48.0%)**	**57 (55.9%)**	
**Median CD34**^**+**^**cells per graft, 10**^**6**^**/kg (range)**	**6.3 (3.7–10.2)**	**6.1 (3.3–11.3)**	**0.746**

*Abbreviations: WBC* white blood cell, *NPM1* nucleophosmin1, *EBV* Epstein-Barr virus, *D* donor, *R* recipient, *CMV* cytomegalovirus, *CR* complete remission, *CR2* second complete remission, *CR1* first complete remission, *MRD* minimal residual disease, *GVHD* graft-versus-host disease, *CsA* cyclosporine A, *MTX* methotrexate, *MMF* mycophenolate, *ATG* antithymocyte immunoglobulin, *MSD* HLA-matched sibling donor, *MUD* HLA-matched unrelated donor, *HID* haploidentical donor, *PBSC* peripheral blood stem cell, *BM* bone marrow. ^#^ Cytogenetic risk was determined according to ELN 2010 criteria

### EBV-DNAemia and EBV-associated diseases

**Fig. 2:**
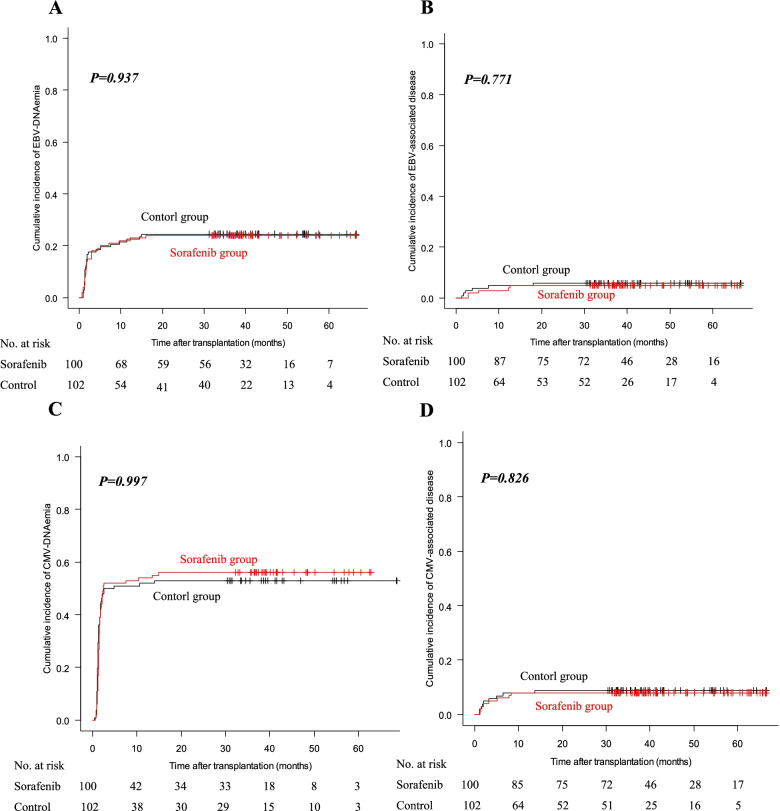
Cumulative incidences of EBV-DNAemia (**A**), EBV-associated disease (**B**), CMV-DNAemia (**C**), and CMV-associated disease (**D**) in the sorafenib and control groups

EBV-DNAemia occurred in 22 (22.0%) patients in the sorafenib group and 23 (22.5%) patients in the control group (*P*=0.925). Four patients developed EBV-DNAemia 1 year after allo-HSCT, including two in the sorafenib group and two in the control group. The 1-year cumulative incidence of EBV-DNAemia was 22.0% (95% CI: 14.4–30.6%) and 22.5% (15.0–31.1%) in the sorafenib and control groups (*HR*=0.946, 95% *CI*: 0.527–1.698, *P*=0.931). The 3-year cumulative incidence of EBV-DNAemia was 24.0% (16.1–32.8%) and 24.5% (16.6–33.2%) in the two groups, respectively (*HR*=0.930, 95% *CI*: 0.531–1.629, *P*=0.937) (Fig. 2A).

Five patients developed EBV-associated diseases in the sorafenib group including 4 EBV-post-transplant lymphoproliferative diseases (PTLD) and 1 EBV-pneumonia, and 6 patients in the control group including 3 EBV-PTLD, 2 EBV-enteritis, and 1 EBV-encephalitis. Two patients developed EBV-associated diseases 1 year after allo-HSCT, including one in the sorafenib group and one in the control group. The 1-year cumulative incidence of EBV-associated diseases was 4.0% (95% CI: 1.3–9.2%) and 4.9% (1.8–10.4%) in the sorafenib and control groups (*HR*=0.745, 95% *CI*: 0.200–2.779, *P*=0.744). The 3-year cumulative incidence of EBV-associated diseases was 5.0% (1.8–10.6%) and 5.9% (2.4–11.6%) in the two groups, respectively (*HR*=0.745, 95% *CI*: 0.227–2.448, *P*=0.771) (Fig. 2B).

Only one patient in the control group received EBV-CTL for EBV-PTLD, and none of the patients received DLI for EBV infections. No patients in the sorafenib group died of EBV-associated diseases, and one patient in the control group died of EBV-PTLD. The 3-year cumulative mortality of EBV-associated diseases was 0.0% (95% CI: 0.0–0.0%) and 1.0% (0.1–4.8%) in the sorafenib and control groups (*HR*=0.016, 95% *CI*: 0.0–150458.5, *P*=0.322).

### CMV-DNAemia and CMV-associated diseases

CMV-DNAemia occurred in 54 (54.0%) patients in the sorafenib group and 53 (52.0%) patients in the control group (*P*=0.772). Seven patients developed CMV-DNAemia 100 days after allo-HSCT, including four in the sorafenib group and three in the control group. The initial and maximum CMV loads in the sorafenib group were 2070 (range, 512–33,600) copies/ml and 2750 (range, 550–175,000) copies/ml, compared with 1790 (range, 550–12,300) copies/ml and 3120 (range, 570–70,500) copies/ml in the control group (*P*=0.612; *P*=0.882). The duration of CMV-DNAemia was 16 (range, 4–50) days and 17 (range, 4–77) days in the sorafenib and control groups (*P*=0.904). The 1-year cumulative incidence of CMV-DNAemia was 54.0% (95% CI: 43.7–63.2%) and 52.0% (41.8–61.2%) in the sorafenib and control groups (*HR*=0.974, 95% *CI*: 0.667–1.423, *P*=0.911). The 3-year cumulative incidence of CMV-DNAemia was 56.0% (45.6–65.1%) and 52.9% (42.7–62.1%) in the two groups, respectively (*HR*=0.991, 95% *CI*: 0.682–1.441, *P*=0.997) (Fig. 2C).

Up to the last follow-up, 8 patients developed CMV-associated diseases in the sorafenib group including 4 CMV-pneumonia, 3 CMV-enteritis, and 1 CMV-retinitis, and 9 patients in the control group including 6 CMV-enteritis, 2 CMV-pneumonia, and 1 CMV-encephalitis. Six patients developed CMV-associated diseases 100 days after allo-HSCT, including three in the sorafenib group and three in the control group. The 1-year cumulative incidence of CMV-associated diseases was 8.0% (95% CI: 3.7–14.4%) and 7.8% (3.7–14.1%) in the sorafenib and control groups (*HR*=0.949, 95% *CI*: 0.356–2.531, *P*=0.984). The 3-year cumulative incidence of CMV-associated diseases was 8.0% (3.7–14.4%) and 8.8% (4.3–15.3%) in the two groups, respectively (*HR*=0.830, 95% *CI*: 0.320–2.155, *P*=0.826) (Fig. 2D).

Seven patients (four in the sorafenib group and three in the control group) received CMV-CTL for CMV infections, and none of the patients received DLI for CMV infections. Two patients in the sorafenib group and two in the control group died of CMV-associated diseases. The 3-year cumulative mortality of CMV-associated diseases was 2.0% (95% CI: 0.4–6.4%) and 2.0% (0.4–6.3%) in the sorafenib and control groups (*HR*=0.955, 95% *CI*: 0.134–6.786, *P*=0.980).

### Risk factors for EBV and CMV infections

Univariable and multivariable analyses of the risk factors for EBV and CMV infections post-transplantation are shown in Tables 2 and 3. All patients undergoing HID/MUD transplants received ATG as GVHD prophylaxis, and none of patients undergoing MSD transplants received ATG as GVHD prophylaxis. Considering there was collinearity between transplant modality (HID/MUD vs MSD) and ATG use in the conditioning (ATG vs no ATG), we only included ATG use in the analysis of risk factors for EBV/CMV infections. On multivariate analysis, ATG use was the only risk factor for EBV-DNAemia (*HR*=4.408, 95% *CI*: 1.967–9.878, *P*<0.001) and EBV-associated diseases (*HR* =3.235, 95% *CI*: 1.078–9.711, *P*=0.036), respectively. ATG use (*HR* =2.797, 95% *CI*: 1.783–4.387, *P*<0.001) and aGVHD (*HR* =1.641, 95% *CI*: 1.067–2.522, *P*=0.024) were the risk factors for CMV-DNAemia; aGVHD (*HR* =3.179, 95% *CI*: 1.175–8.601, *P*=0.023) was the only risk factor for CMV-associated diseases. In contrast, age, sex, EBV and CMV serological status, CR status at transplantation, sorafenib use pre-transplantation and post-transplantation, and cGVHD did not show any significant influence on the risk of EBV and CMV infections.

**Table 2:** Univariable analysis for the risk factors of EBV and CMV infections post-transplantation

Parameters	EBV-DNAemia	EBV-associated disease	CMV-DNAemia	CMV-associated disease
**Gender**	***P*=0.679**	***P*=0.125**	***P*=0.385**	***P*=0.844**
**Female vs male**				
**Patient age**	***P*=0.687**	***P*=0.881**	***P*=0.137**	***P*=0.694**
**< 35 vs ≥ 35 years**				
**EBV serostatus**	***P*=0.067**	***P*=0.502**	**--**	**--**
**D−/R+ vs other**				
**CMV serostatus**	**--**	**--**	***P*=0.573**	***P*=0.797**
**D−/R+ vs other**				
**CR status at transplant**	***P*=0.163**	***P*=1.000**	*P=0.025	***P*=0.455**
**≥ CR2 vs CR1**				
**ATG use in the conditioning**^#^	*P<0.001	*P=0.036	*P<0.001	***P*=0.985**
**ATG vs no ATG**				
**Sorafenib pre-transplant**	***P*=0.349**	***P*=0.727**	***P*=0.085**	***P*=0.296**
**Use vs no use**				
**Sorafenib post-transplant**	***P*=0.853**	***P*=0.628**	***P*=0.892**	***P*=0.702**
**Sorafenib vs control**				
**aGVHD**^&^	***P*=0.417**	***P*=0.196**	*P=0.004	*P=0.023
**cGVHD**^&^	***P*=0.891**	***P*=0.190**	***P*=0.248**	***P*=0.120**

*Abbreviations: EBV* Epstein-Barr virus, *D* donor, *R* recipient, *CMV* cytomegalovirus, *CR* complete remission, *CR2* second CR, *CR1* first CR, *ATG* antithymocyte immunoglobulin; *aGVHD*, acute graft-versus-host disease; *cGVHD*, chronic GVHD. *P<0.05. ^#^All patients undergoing haploidentical donor (HID)/HLA-matched unrelated donor (MUD) transplants received ATG as GVHD prophylaxis, and none of those undergoing HLA-matched sibling donor (MSD) transplants received ATG as GVHD prophylaxis. Considering there was collinearity between transplant modality (HID/MUD vs MSD) and ATG use in the conditioning (ATG vs no ATG), we only included ATG use in the conditioning in the analysis of risk factors for EBV/CMV infections. ^&^Time-dependent covariate

**Table 3:** Multivariable analysis for the risk factors of EBV and CMV infections post-transplantation

Parameters	HR	95% CI	***P*** value
**EBV-DNAemia**			
**EBV serostatus: D- /R+ vs other**	**2.142**	**0.956–4.797**	**0.064**
**ATG use in the conditioning: ATG vs no ATG**^#^	**4.408**	**1.967–9.878**	*<0.001
**EBV-associated disease**			
**ATG use in the conditioning: ATG vs no ATG**^#^	**3.235**	**1.078–9.711**	*0.036
**CMV-DNAemia**			
**CR status at transplant: ≥ CR2 vs CR1**	**1.222**	**0.931–1.603**	**0.149**
**ATG use in the conditionin: ATG vs no ATG**^#^	**2.797**	**1.783–4.387**	*<0.001
**Sorafenib pre-transplant: use vs no use**	**1.340**	**0.910–1.974**	**0.138**
**aGVHD**^&^	**1.641**	**1.067–2.522**	*0.024
**CMV-associated disease**			
**aGVHD**^&^	**3.179**	**1.175–8.601**	*0.023

*Abbreviations: EBV* Epstein-Barr virus, *D* donor, *R* recipient, *ATG* antithymocyte immunoglobulin, *CMV* cytomegalovirus, *CR* complete remission, *CR2* second CR, *CR1* first CR, *aGVHD* acute graft-versus-host disease, *HR* hazard ratio, *CI* confidence interval. *P<0.05; ^#^ All patients undergoing haploidentical donor (HID)/HLA-matched unrelated donor (MUD) transplants received ATG as GVHD prophylaxis, and none of those undergoing HLA-matched sibling donor (MSD) transplants received ATG as GVHD prophylaxis. Considering there was collinearity between transplant modality (HID/MUD vs MSD) and ATG use in the conditioning (ATG vs no ATG), we only included ATG use in the conditioning in the analysis of risk factors for EBV/CMV infections. ^&^Time-dependent covariate

### Immune reconstitution

Immune reconstitution was similar with respect to the counts of T lymphocyte subsets (CD3^+^, CD3^+^CD4^+^, CD3^+^CD8^+^), B lymphocytes (CD19^+^), and NK cells (CD3^-^CD56^+^) at 1, 3, 6, 9, and 12 months after allo-HSCT between the sorafenib and control groups (all *P* >0.05) (Table 4).

**Table 4:** Immune reconstitution within 1 year post-transplantation (absolute value)

		CD3^**+**^	CD3^**+**^CD4^**+**^	CD3^**+**^CD8^**+**^	CD19^**+**^	CD3^**-**^CD56^**+**^
Months after allo-HSCT		Mean absolute value (10^9^/L, range)	Mean absolute value (10^9^/L, range)	Mean absolute value (10^9^/L, range)	Mean absolute value (10^9^/L, range)	Mean absolute value (10^9^/L, range)
**1**^**st**^**(*N*=202)**	**Sorafenib group**	0.450 (0.025–1.218)	0.086 (0.007**–**0.239)	0.296 (0.009**–**0.938)	0.009 (0.000**–**0.049)	0.137 (0.024**–**0.336)
	**Control group**	0.621 (0.047**–**1.515)	0.085 (0.007**–**0.144)	0.451 (0.032**–**1.392)	0.010 (0.001**–**0.035)	0.193 (0.079**–**0.378)
	***P***	*0.267*	*0.966*	*0.262*	*0.870*	*0.159*
**3**^**rd**^**(*N*=188)**	**Sorafenib group**	0.985 (0.191**–**2.835)	0.182 (0.030**–**0.470)	0.681 (0.077**–**2.446)	0.059 (0.000**–**0.336)	0.323 (0.026**–**0.910)
	**Control group**	1.294 (0.510**–**3.779)	0.209 (0.057**–**0.485)	0.977 (0.183**–**3.285)	0.039 (0.000**–**0.102)	0.275 (0.102**–**0.534)
	***P***	*0.261*	*0.501*	*0.244*	*0.416*	*0.519*
**6**^**th**^**(*N*=165)**	**Sorafenib group**	1.273 (0.398**–**2.823)	0.257 (0.105**–**0.674)	0.863 (0.202**–**2.633)	0.082 (0.002**–**0.307)	0.272 (0.095**–**0.595)
	**Control group**	1.228 (0.322**–**2.925)	0.232 (0.105**–**0.347)	0.907 (0.122**–**2.500)	0.076 (0.007**–**0.226)	0.246 (0.098**–**0.529)
	***P***	*0.858*	*0.824*	*0.587*	*0.557*	*0.842*
**9**^**th**^**(*N*=152)**	**Sorafenib group**	1.394 (0.211**–**3.714)	0.211 (0.058**–**0.458)	1.075 (0.115**–**3.486)	0.111 (0.001**–**0.344)	0.281 (0.025**–**1.069)
	**Control group**	1.236 (0.356**–**3.702)	0.234 (0.055**–**0.546)	0.899 (0.209**–**3.159)	0.137 (0.002**–**0.478)	0.309 (0.052**–**0.903)
	***P***	*0.542*	*0.575*	*0.469*	*0.442*	*0.747*
**12**^**th**^**(*N*=141)**	**Sorafenib group**	1.626 (0.309**–**3.906)	0.293 (0.066**–**0.743)	1.220 (0.222**–**3.630)	0.184 (0.002**–**0.558)	0.271 (0.010**–**0.755)
	**Control group**	1.448 (0.536**–**2.735)	0.315 (0.102**–**0.764)	1.025 (0.333**–**2.494)	0.229 (0.003**–**1.208)	0.370 (0.058**–**1.075)
	***P***	*0.459*	*0.627*	*0.410*	*0.448*	*0.141*

### Transplant outcomes

**Fig. 3:**
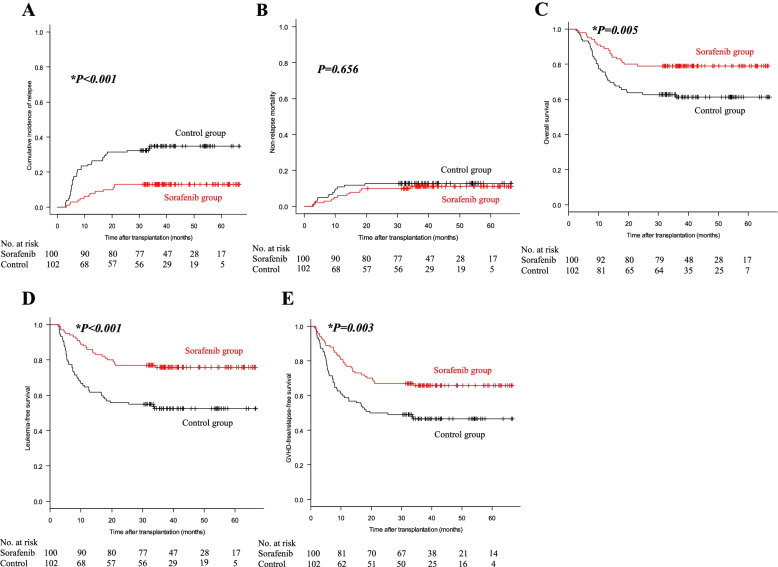
Cumulative incidences of leukemia relapse (**A**), non-relapse mortality (**B**), overall survival (**C**), leukemia-free survival (**D**), and GVHD-free/relapse-free survival (**E**) in the sorafenib and control groups. *P < 0.05

At the date of statistical analysis, 142 patients survived and 60 died, of whom 21 were in the sorafenib group and 39 were in the control group. Causes of death were leukemia relapse (*n*=31; 7 in the sorafenib group and 24 in the control group), infections (*n*=18; 10 in the sorafenib group and 8 in the control group), GVHD (*n*=8; 3 in the sorafenib group and 5 in the control group), EBV-PTLD (*n*=1; control group), thrombotic microangiopathy (*n*=1; control group), and acute left heart failure (*n*=1; sorafenib group). The 3-year cumulative incidence of relapse was 13.0% (95% CI: 7.3–20.4%) and 34.8% (25.5–44.2%) in the sorafenib and control groups, respectively (*HR*=0.306, 95% *CI*: 0.162–0.579, *P*<0.001) (Fig. 3A). The 3-year NRM was 11.1% (95% CI: 5.9–18.3%) and 12.7% (7.1–20.0%) in the two groups (*HR*=0.689, 95% *CI*: 0.308–1.540, *P*=0.656) (Fig. 3B). The 3-year OS was 79.0% (95% CI: 69.6–85.8%) and 61.4% (51.1–70.1%; *HR*=0.481, 95% *CI*: 0.283–0.818, *P*=0.005), LFS was 75.9% (95% CI: 66.2–83.1%) and 52.5% (42.2–61.7%; *HR*=0.410, 95% *CI*: 0.251–0.670, *P*<0.001), and GRFS was 65.8% (95% CI: 55.6–74.3%) and 46.6% (36.6–56.0%; *HR*=0.531, 95% *CI*: 0.345–0.816, *P*=0.003), respectively, in the sorafenib and control groups (Fig. 3C–E).

## Discussion

In this study, we investigated the effect of sorafenib maintenance post-transplantation on EBV and CMV infections. Our study showed that sorafenib maintenance post-transplantation did not increase the incidence and mortality of EBV and CMV-associated diseases compared with those with non-maintenance post-transplantation. Besides, after prolonged follow-up of 36.8 months, our data once again proved that sorafenib maintenance post-transplantation could reduce relapse and improve survival.

With the widespread use of kinase inhibitors in oncology patients, the risk of opportunistic viral infections in the patients receiving kinase inhibitors has been reported [11–20]. Some studies revealed that dasatinib might reactivate latent CMV infection and induce CMV-associated diseases, such as CMV pneumonitis, colitis, and hepatitis [11, 12, 15, 19]. Case reports from Japan reported the patients with chronic myeloid leukemia who developed EBV-lymphoproliferative disease during imatinib and dasatinib treatment, respectively [13, 14]. PDGFR-ɑ has been reported as a critical receptor required for CMV infection [30]. Interestingly, a recent phase 2 study showed that nilotinib could prevent CMV infection after allo-HSCT by inhibiting PDGFR- ɑ[31]. However, this study was a single-arm study with a small sample size. It was difficult to draw definite conclusions from the results, which required to be verified by multi-center RCTs. With regard to the effect of sorafenib on EBV or CMV infections, Park et al. reported that sorafenib induced apoptosis of EBV-transformed B cells through ROS-dependent JNK/p38-MAPK signaling in an ERK-independent manner [21]. Michaelis et al. demonstrated that sorafenib diminished human CMV replication in clinically relevant concentrations and inhibited human CMV immediate early antigen expression through inhibition of Raf [22]. However, prospective or retrospective studies about the effect of sorafenib on EBV or CMV infections in sorafenib-treated patients are lacking. In this large-sample data from RCT, the results revealed that sorafenib maintenance post-transplantation did not increase the incidence of EBV and CMV-DNAemia as well as the incidence and mortality of EBV and CMV-associated diseases, compared with those with non-maintenance post-transplantation. Our result also revealed that the 3-year NRM was similar between the two groups. These results further suggested that sorafenib maintenance post-transplantation was safe and well tolerated by patients with FLT3-ITD AML.

Multiple factors could influence the occurrence of EBV and CMV infections, such as T-cell depletion, use of ATG, aGVHD, donor other than MSD, and serological status of donor and recipient [25, 32–34]. Our results accorded with previous work, showing that use of ATG was the risk factor for EBV-DNAemia, CMV-DNAemia, and EBV-associated diseases; aGVHD was the risk factor for CMV-DNAemia and CMV-associated diseases. CMV serological status was not identified as a risk factor for CMV infections, which might be due to that more than 80% patients and their donors were CMV-seropositive in this study. Multivariable analysis also showed that sorafenib use pre-transplantation and post-transplantation were not the risk factors for EBV/CMV-DNAemia and EBV/CMV-associated diseases. The results confirmed that sorafenib maintenance post-transplantation did not increase the risk of EBV and CMV infections.

The occurrence of EBV and CMV infections is closely related to the immune function of recipients [25, 35, 36]. Some studies have revealed that use of kinase inhibitors might change the expression and function of immune cells [18, 37–39]. Seggewiss et al. reported that imatinib inhibited T-cell receptor-mediated T-cell proliferation and activation, and CMV- and EBV-specific CD8^+^ T cell responses were also suppressed by imatinib [39]. Interestingly, administration of sorafenib might contribute to increase infiltration of activated CD8^+^ T cells. Mathew et al. showed that sorafenib promoted CD8^+^ T cell activation and graft-versus-leukemia activity through interleukin-15 production [37]. Kalathil et al. demonstrated that augmentation of IFN-γ^+^CD8^+^ T cell responses correlated with survival of patients with hepatocellular carcinoma who were treated with sorafenib [38]. In this study, we compared the immune reconstitution of the sorafenib and control groups at 1, 3, 6, 9, and 12 months after allo-HSCT. Our results showed that the reconstitution of T lymphocyte subsets includingCD3^+^CD8^+^ T cells, B lymphocytes, and NK cells were similar between the two groups. Cell therapy such as CTL or DLI could affect the immune reconstitution of recipients post-transplantation. Given that the number of patients receiving CTL or DLI for EBV/CMV infections was small and similar between the two groups, we thought that the number of patients who received CTL or DLI might not affect the difference in immune reconstitution between the sorafenib and control groups.

Early results of our previous RCT after a median follow-up of 21.3 months showed that sorafenib maintenance post-transplantation led to a significant improvement in relapse and survival in patients with FLT3-ITD AML [6]. Here we present results after prolonged follow-up of 36.8 months, demonstrating the anti-leukemic efficacy of sorafenib maintenance post-transplantation in reducing relapse and prolonging survival. Additionally, we found that sorafenib maintenance post-transplantation had improved GRFS compared with non-maintenance.

Our study had some limitations. First, it was not a RCT which was designed to compare the effect of sorafenib maintenance post-transplantation on EBV and CMV infections. Second, we did not conduct the detections of EBV- and CMV-CTL reconstitutions post-transplantation in this study.

## Conclusions

Sorafenib maintenance post-transplantation does not increase the incidence and mortality of EBV and CMV infections, demonstrating a favorable safety profile.

## Supplementary Information

**Additional file 1.** Study protocol.

## Data Availability

The datasets used and analyzed during the current study are available from the corresponding author on reasonable request.

## Abbreviations

aGVHD: Acute GVHD
allo-HSCT: Allogeneic hematopoietic stem cell transplantation
AML: Acute myeloid leukemia
ATG: Antithymocyte immunoglobulin
BM: Bone marrow
cGVHD: Chronic GVHD
CMV: Cytomegalovirus
CR: Complete remission
CsA: Cyclosporine A
CTL: Cytotoxic T lymphocyte
DLI: Donor lymphocyte infusion
EBV: Epstein-Barr virus
FLT3-ITD: FLT3 internal tandem duplication
GRFS: GVHD-free/relapse-free survival
GVHD: Graft-versus-host disease
HID: Haploidentical donor
HR: Hazard ratio
IFI: Invasive fungal infection
LFS: Leukemia-free survival
MMF: Mycophenolate
MSD: HLA-matched sibling donor
MTX: Methotrexate
MUD: HLA-matched unrelated donor
NK: Natural killer
NRM: Non-relapse mortality
OS: Overall survival
PB: Peripheral blood
PBSC: Peripheral blood stem cell
PCR: Polymerase chain reaction
PDGFR: Platelet-derived growth factor receptor
PTLD: Post-transplant lymphoproliferative disease
RCT: Randomized controlled trial
