# Supplementary material for: Effect of sorafenib maintenance on Epstein-Barr virus and cytomegalovirus infections in patients with FLT3-ITD AML undergoing allogeneic hematopoietic stem cell transplantation: a secondary analysis of a randomized clinical trial
Source: BMC Med. 2022 Sep 2;20:282. doi: 10.1186/s12916-022-02479-x (PMC9436457; doi:10.1186/s12916-022-02479-x)
Supplement: Supplementary file 1 — Additional file 1. Study protocol. [file 12916_2022_2479_MOESM1_ESM.docx]

**CLINICAL STUDY PROTOCOL**

**Protocol Title: A prospective, open-label, multicenter,** **randomized, phase 3 study to assess the efficacy and tolerability of sorafenib as** **maintenance therapy in FLT3-ITD acute myeloid leukemia undergoing allogeneic hematopoietic stem cell transplantation**

**Protocol Number: NFEC-2015-049**

**Study Drug: sorafenib**

**Study Phase: 3**

**Indication: FLT3-ITD acute myeloid leukemia undergoing allogeneic hematopoietic stem cell transplantation**

**Applicant Institution:** **Nanfang Hospital, Southern Medical University**

**Principal institution: Nanfang Hospital, Southern Medical University**

**Version: 2.0**

**Date: 20150630**

**Remark: the primary version of this protocol was in Chinese. We have translated it into English.**

**INVESTIGATOR'S STATEMENT**

I have received and completely reviewed the following protocol (NFEC-2015-049, Protocol Version 2.0, dated 30 June 2015), including all appendices:

As Principal Investigator, I understand and agree to conduct this clinical study as described and will comply with the ethical and regulatory considerations delineated herein.

**Study Title**

A prospective, open-label, multicenter, randomized, phase 3 study to assess the efficacy and tolerability of sorafenib as maintenance therapy in FLT3-ITD acute myeloid leukemia undergoing allogeneic hematopoietic stem cell transplantation

**Principal Investigator Signature and Contact Information**

**Principal Investigator (print)**

**Principal Investigator (signature)**

**Date of Signature**

**Institution/Affiliation**

**City, Province, Country**

# Study Synopsis

| **Study title** | A prospective, open-label, multicenter, randomized, phase 3 study to assess the efficacy and tolerability of sorafenib as maintenance therapy in FLT3 internal tandem duplication (FLT3-ITD) acute myeloid leukemia (AML) undergoing allogeneic hematopoietic stem cell transplantation (allo-HSCT) |
| --- | --- |
| **Protocol number** | NFEC-2015-049 |
| **Indication** | FLT3-ITD AML undergoing first allo-HSCT |
| **Study phase** | 3 |
| **Study applicant** | Nanfang Hospital, Southern Medical University |
| **Study centers** | 7 study centers (Nanfang Hospital, Peking University People's Hospital, Xiangya Hospital, Zhujiang Hospital, Third Affiliated Hospital of Sun Yat-Sen University, First People’s Hospital of Chenzhou, First Affiliated Hospital of Guangzhou Medical University) |
| **Number of subjects planned** | Approximately 194 subjects (97 each group) will be randomized. |
| **Study duration** | Estimated to be 3 years |
| **Objectives** | **Primary objective:**  To compare the 1-year cumulative incidence of relapse in patients with FLT3-ITD AML undergoing allo-HSCT who receive sorafenib maintenance versus non-maintenance post-transplantation.  **Secondary objectives:**  To compare overall survival (OS), leukemia-free survival (LFS) and adverse effects (AEs) in patients with FLT3-ITD AML undergoing allo-HSCT who receive sorafenib maintenance versus non-maintenance post-transplantation. |
| **Study design** | This is a prospective, open-label, multicenter, randomized, phase 3 study comparing sorafenib maintenance with non-maintenance post-transplantation for patients with FLT3-ITD AML undergoing allo-HSCT. Approximately 194 subjects will be randomized in a 1:1 ratio to receive sorafenib maintenance (97 subjects) or non-maintenance (97 subjects) post-transplantation. Randomization is done with permuted blocks (block size four), and implemented through an interactive web-based response system.  **Sorafenib Group:** Sorafenib maintenance (Sorafenib is administrated at 30-60 days post-transplantation and continued until day +180).  **Control Group:** Non-maintenance (Neither sorafenib nor other FLT3 inhibitors are used post-transplantation).  Disease assessment including routine blood and bone marrow (BM) assessment will be regularly performed post-transplantation. BM assessment, including BM smear and FLT3-ITD mutation is performed before randomization, every month for the first three months after enrollment, every two months from the 4th to 9th month after enrollment, and then every three months until the study is completed.  All subjects will be followed for safety and tolerability within 210 days post-transplantation. With the exception of graft-versus-host disease (GVHD) and hematologic AEs, all AEs are graded according to CTCAE version 4.0. Acute GVHD (aGVHD), chronic GVHD (cGVHD) and hematologic AEs are graded as AEs according to our self-defined criteria. |
| **Inclusion criteria** | Subjects eligible for enrolment in this study must meet all of the following criteria:   1. Patients with FLT3-ITD AML undergoing first allo-HSCT 2. Age 18 to 60 years old with Eastern Cooperative Oncology Group (ECOG) performance status 0-2 3. Composite complete remission (CRc) before and after allo-HSCT (CRc must be confirmed by BM analysis within 3 days before randomization)  - CRc comprises complete remission (CR), CR with incomplete platelet recovery (CRp), and CR with incomplete hematologic recovery (CRi). - CR is defined as bone marrow (BM) blasts <5%; absence of circulating blasts and blasts with Auer rods; absence of extramedullary disease; absolute neutrophil count (ANC) ≥1·0×10^9^/L and platelet count (PLT) ≥100×10^9^/L. - CRp is defined as all CR criteria except for incomplete platelet recovery (<100×10^9^/L). - CRi is defined as all CR criteria except for incomplete hematologic recovery with residual neutropenia <1·0×10^9^/L and/ or thrombocytopenia <100×10^9^/L.  1. Hematopoietic recovery within 60 days post-transplantation  - Hematopoietic recovery post-transplantation is defined as an ANC ≥ 1.0 × 10^9^/L and a non-transfused PLT ≥ 30 × 10^9^/L within three days.  1. Sign informed consent form, have the ability to comply with study and follow-up procedures |
| **Exclusion criteria** | Subjects meeting any of the following criteria are ineligible for this study:   1. Acute promyelocytic leukemia (AML subtype M3) 2. Intolerance to sorafenib pre-transplantation 3. Life expectancy less than 30 days post-transplantation 4. Active aGVHD or uncontrolled infections within 60 days post-transplantation 5. Cardiac dysfunction (particularly congestive heart failure, unstable coronary artery disease and serious cardiac ventricular arrhythmias requiring antiarrhythmic therapy) 6. Respiratory failure ( PaO2 ≤60mmHg) 7. Hepatic abnormalities (total bilirubin ≥2 times the upper limit of normal [ULN], alanine aminotransferase or aspartate aminotransferase ≥2 times the ULN) 8. Renal dysfunction (creatinine ≥2 times the ULN or creatinine clearance rate < 30 mL/min) 9. ECOG performance status 3, 4 or 5 10. With any conditions not suitable for the trial (investigators' decision) |
| **Study treatment** | **Sorafenib maintenance**  For subjects randomized to sorafenib maintenance (sorafenib group), sorafenib is started from day +30 until day +180 post-transplantation if patients meet the inclusion criterion on day +30. For patients who do not meet the inclusion criterion on day +30 but who do so within 31-60 days post-transplantation, sorafenib is administrated at 31-60 days post-transplantation and continued until day +180. The initial dose of sorafenib is 400 mg orally twice daily (BID). In cases of grade 3 or worse AEs, dose modifications including dose reductions or interruptions are allowed at the investigator’s discretion. After the resolution of AEs, the dose is re-escalated from 200 mg once every day or 200 mg BID to 400 mg BID.  **Non-maintenance**  For subjects randomized to non-maintenance (control group), neither sorafenib nor other FLT3 inhibitors are used unless the patients experience relapse. |
| **Sample size determination** | This trial is designed to test the hypothesis that sorafenib maintenance post-transplantation is superior to non-maintenance post-transplantation with respect to relapse. The sample size calculation is based on the primary endpoint, the 1-year cumulative incidence of relapse, which is approximately 40% in the patients with FLT3-ITD AML undergoing allo-HSCT with non-maintenance post-transplantation. To identify a 20% absolute decrease in the 1-year cumulative incidence of relapse with sorafenib maintenance post-transplantation, a minimum of 176 patients (88 in each group) is required to provide the study with a significance level of 0.05 and a power of 90%. After adjusting for a 10% dropout, the total planned sample size is 194 patients (97 in each group). |
| **Statistical analysis** | Statistical analysis is performed based on the intent-to-treat (ITT) population, which includes all randomized subjects.  **Primary Efficacy Analysis:**  The primary endpoint of this study is the 1-year cumulative incidence of relapse post-transplantation, which is calculated by accounting for competing risk. Non-relapse mortality is considered a competing risk for relapse. The comparison of the cumulative incidence of relapse is tested using Fine and Gray model.   - Relapse is defined as reappearance of leukemic blasts in the peripheral blood or ≥ 5% blasts in the BM as pirate or biopsy not attributable to any other cause or reappearance or new appearance of extramedullary leukemia.   **Secondary Efficacy Analysis:**  The secondary endpoints of OS and LFS are estimated using the Kaplan-Meier method and compared using the log-rank test.   - OS is defined as the time from transplantation until death from any cause. - LFS is defined as survival in continuous CR without relapse, and refer to the time from transplantation until relapse or death from any cause.   **Safety Analysis:**  Safety and tolerability will be assessed by incidence and severity of AEs and changes from baseline of all relevant parameters, including laboratory test values, physical examination, vital signs, and ECOG performance scores. With the exception of GVHD and hematologic AEs, all AEs are graded according to CTCAE version 4.0. aGVHD, cGVHD and hematologic AEs are graded as AEs according to our self-defined criteria. All subjects will be monitored for AEs within 210 days post-transplantation. |
|  |  |

**TABLE OF CONTENTS**

PAGE

[Clinical Study Protocol 1](#_Toc512010108)

[Investigator's Statement 2](#_Toc512010108)

[Study Synopsis 3](#_Toc512010108)

[Table of Contents 9](#_Toc512010108)

[Abbreviations 11](#_Toc512010108)

[1. Introduction 13](#_Toc512010109)

[2. Study objectives 14](#_Toc512010110)

[2.1 Primary Objective 14](#_Toc512010111)

[2.2 Secondary Objectives 14](#_Toc512010112)

[3. Study Design 14](#_Toc512010113)

[4. Subject Selection Criteria 16](#_Toc512010114)

[4.1 Subject Selection Criteria 16](#_Toc512010115)

[4.1.1 Number of Subjects 16](#_Toc512010116)

[4.1.2 Inclusion Criteria 17](#_Toc512010117)

[4.1.3 Exclusion Criteria 17](#_Toc512010118)

[4.2.](#_Toc512010119) [Withdrawal Criteria 18](#_Toc512010120)

[5. Study Procedures 19](#_Toc512010121)

[5.1 Screening 19](#_Toc512010122)

[5.2 Treatment Allocation and Blinding 19](#_Toc512010122)

[5.3 Study Treatment 19](#_Toc512010123)

[5.3.1 Sorafenib Group (sorafenib maintenance therapy) 19](#_Toc512010123)

[5.3.2 Control Group (non-maintenance therapy) 2](#_Toc512010123)0

[5.4 Follow-up 2](#_Toc512010123)0

[6. Efficacy Assessments 2](#_Toc512010124)0

[6.1 Definitions 2](#_Toc512010126)0

[6.2 Primary Efficacy Endpoint 21](#_Toc512010127)

[6.3 Secondary Efficacy Endpoints 21](#_Toc512010128)

[6.4 Schedule and methods of Efficacy Assessments 21](#_Toc512010128)

[7. Safety Evaluation 22](#_Toc512010129)

[7.1 Medical History 22](#_Toc512010130)

[7.2 Vital Signs and Physical Examination 22](#_Toc512010131)

[7.3 Clinical Symptoms 23](#_Toc512010131)

[7.4 Clinical Laboratory Evaluations 23](#_Toc512010132)

[8. Adverse Events and Serious Adverse Events (SAEs) 24](#_Toc512010134)

[8.1 Definitions 24](#_Toc512010135)

[8.1.1 Adverse Events 24](#_Toc512010136)

[8.1.2 Serious Adverse Events 24](#_Toc512010137)

[8.2 Assessment of Severity 25](#_Toc512010131)

[8.3 Assessment of Causality 26](#_Toc512010131)

[8.4 Recording and Reporting AEs and SAEs 26](#_Toc512010131)

[9. Rules of Withdrawal 27](#_Toc512010134)

[9.1 Subjects Withdraw from the Study 27](#_Toc512010135)

[9.2 Premature Termination of the Study 27](#_Toc512010136)

[10. Rules of Follow-Up 28](#_Toc512010134)

[10.1 Follow-up Period 28](#_Toc512010135)

[10.2 Visit Scheduling 28](#_Toc512010136)

[10.3 Contents 28](#_Toc512010137)

[11. Data Analysis and Statistical Considerations 28](#_Toc512010138)

[11.1 Hypotheses 28](#_Toc512010139)

[11.2 Study Design Considerations 29](#_Toc512010139)

[11.2.1 Sample Size Assumptions 29](#_Toc512010140)

[11.2.2 Primary Efficacy Endpoint 29](#_Toc512010141)

[11.2.3 Secondary Efficacy Endpoints 3](#_Toc512010142)0

[11.3 Data Analysis Considerations 3](#_Toc512010143)0

[11.3.1 Analysis Population 3](#_Toc512010144)0

[11.3.2](#_Toc512010145) [Analysis Plan 3](#_Toc512010144)0

[11.3.2.1 Baseline Data 3](#_Toc512010145)0

[11.3.2.2 Analysis of Efficacy 3](#_Toc512010144)0

[11.3.2.3 Analysis of Safety 3](#_Toc512010145)1

[12. Materials for the Study 31](#_Toc512010146)

[13. Ethical Considerations 31](#_Toc512010147)

[13.1 Responsibility of Investigators 31](#_Toc512010148)

[13.2 Informed Consent Process 31](#_Toc512010149)

[13.3 Good Clinical Practice 32](#_Toc512010149)

[13.4 Protection of Subjects’ Personal Data 32](#_Toc512010150)

[14. Administrative Requirements 32](#_Toc512010151)

[15. References 33](#_Toc512010151)

[16. Appendices 35](#_Toc512010152)

[16.1 Appendix 1 Diagnosis and Classification of aGVHD and cGVHD 35](#_Toc512010155)

16.2 Appendix 2 ECOG Performance Satus 40

**Abbreviations**

| ADL | Activities of Daily Living |
| --- | --- |
| AE | Adverse Event |
| aGVHD | Acute Graft-versus-Host Disease |
| Allo-HSCT | Allogeneic Hematopoietic Stem Cell Transplantation |
| ALT | Alanine Aminotransferase |
| AML | Acute Myeloid Leukemia |
| AST | Aspartate Aminotransferase |
| BID | Twice Daily |
| BM | Bone Marrow |
| cGVHD | Chronic Graft-versus-Host Disease |
| CR | Complete Remission |
| CRF | Case Report Form |
| CTCAE | Common Terminology Criteria for Adverse Events |
| ECOG | Eastern Cooperative Oncology Group |
| FLT3-ITD | FMS-Like Tyrosine Kinase 3-Internal Tandem Duplication |
| GCP | Good Clinical Practice |
| GVHD | Graft-versus-Host Disease |
| ITT | Intent-To-Treat |
| LFS | Leukemia-Free Survival |
| NIH | National Institutes of Health |
| NR | No Evidence of Response |
| OS | Overall Survival |
| PB | Peripheral Blood |
| PDGF | Platelet-Derived Growth Factor |
| PR | Partial Remission |
| QD | Once Every Day |
| SAE | Serious Adverse Event |
| TKD | Tyrosine Kinase Domain |
| ULN | Upper Limit of Normal |
| VEGF | Vascular Endothelial Growth Factor |

# 1. Introduction

Acute myeloid leukemia (AML) is a heterogeneous tumor derived from hematopoietic stem cells with a series of cytogenetic, genetic, and epigenetic abnormalities.[^1^](#_ENREF_1) FMS-like tyrosine kinase 3 (FLT3) gene mutations represent one of the most frequent genetic alterations in AML, with an incidence of approximately 30%.[^2^](#_ENREF_2) There are two categories of FLT3 mutations: internal tandem duplication (ITD) occurring in the juxtamembrane domain of the receptor (FLT3-ITD, approximately 25%) and point mutations resulting in single amino acid substitutions in the activation loop of the tyrosine kinase domain (FLT3-TKD, approximately 5%-7%).[^2^](#_ENREF_2) Patients with FLT3-ITD AML usually have a worse prognosis than those with wild-type FLT3 AML due to shorter remission duration and higher relapse, and these patients are rarely cured by chemotherapy alone.[^3^](#_ENREF_3)^,^ [^4^](#_ENREF_4) Allogeneic hematopoietic stem cell transplantation (allo-HSCT) can improve the outcomes of these patients, but the leukemia relapse rate remains high.[^5^](#_ENREF_5)^,^ [^6^](#_ENREF_6)

FLT3-ITD mutations can render the tyrosine kinase constitutively active and thus result in chronic stimulation of downstream signaling pathways and abnormal proliferation of leukemic cells.[^7^](#_ENREF_7) Inhibition of activated FLT3-ITD is an important therapeutic strategy to treat the active disease and prevent relapse. Sorafenib is a multi-kinase inhibitor that blocks multiple pathways involved in the development and progression of AML, such as FLT3-ITD, Ras/Raf, as well as vascular endothelial growth factor (VEGF) and platelet-derived growth factor (PDGF) receptors.[^7^](#_ENREF_7)^,^ [^8^](#_ENREF_8) A growing body of studies has demonstrated that the addition of sorafenib to chemotherapy could prolong the remission duration of FLT3-ITD AML.[^9-11^](#_ENREF_9) A phase I trial reported that sorafenib maintenance post-transplantation was safe for patients with FLT3-ITD AML undergoing allo-HSCT.[^12^](#_ENREF_12) Several retrospective studies suggested that sorafenib maintenance post-transplantation might reduce relapse and improve survival. [^13^](#_ENREF_13)^,^ [^14^](#_ENREF_14) To date, prospective randomized study on sorafenib as maintenance therapy post-transplantation is lacking. Therefore, we design this open-label, multicenter, randomized, phase 3 study to evaluate the efficacy and tolerability of sorafenib as maintenance therapy in patients with FLT3-ITD AML undergoing allo-HSCT.

# 2. Study objectives

## 2.1 Primary Objective

The primary objective of this study is to compare the 1-year cumulative incidence of relapse post-transplantation in patients with FLT3-ITD AML undergoing allo-HSCT who receive sorafenib maintenance versus non-maintenance post-transplantation.

## 2.2 Secondary Objectives

The secondary objectives of this study are to compare overall survival (OS), leukemia-free survival (LFS) and adverse effects (AEs) in patients with FLT3-ITD AML undergoing allo-HSCT who receive sorafenib maintenance versus non-maintenance post-transplantation.

# 3. Study Design

This is a prospective, open-label, multicenter, randomized, phase 3 study of comparison of sorafenib maintenance versus non-maintenance post-transplantation in patients with FLT3-ITD AML undergoing allo-HSCT. The study design is illustrated in Figure 1.

Subjects with FLT3-ITD AML receiving first allo-HSCT will be screened for eligibility. Medical history evaluation, vital sign, physical examination, Eastern Cooperative Oncology Group (ECOG) performance status, blood and urine sampling for laboratory tests, electrocardiogram, chest imaging examination as well as bone marrow (BM) assessment will be performed to determine study eligibility, all of which must be performed ≤3 days prior to randomization. Eligible subjects will be randomized in a 1:1 ratio to receive sorafenib maintenance or non-maintenance post-transplantation. Randomization is performed with randomization codes generated by a computer-generated randomization system.

Based on the randomization and assignment, the subjects will receive sorafenib maintenance or non-maintenance post-transplantation. For subjects in the experimental intervention arm (sorafenib group), sorafenib is started from day +30 until day +180 post-transplantation if patients meet the inclusion criterion on day +30. For patients who do not meet the inclusion criterion on day +30 but who do so within 31-60 days post-transplantation, sorafenib is administrated at 31-60 days post-transplantation and continued until day +180. The initial dose of sorafenib is 400 mg orally twice daily (BID). In cases of grade 3 or worse AEs, dose modifications including dose reductions or interruptions are allowed at the investigator’s discretion. After the resolution of AEs, the dose is re-escalated from 200 mg once every day (QD) or 200 mg BID to 400 mg BID at the discretion of the investigator. For subjects in the no intervention arm (non-maintenance, control group), neither sorafenib nor other FLT3 inhibitors are used unless the patients experience relapse. No crossover between the two groups before relapse will be allowed.

Disease assessment including routine blood and BM assessment will be regularly performed post-transplantation. BM assessment, including BM smear and FLT3-ITD mutation is performed before randomization, every month for the first three months after enrollment, every two months from the 4th to 9th month after enrollment, and then every three months until the study is completed.

All subjects will be followed for safety and tolerability within 210 days post-transplantation. With the exception of graft-versus-host disease (GVHD) and hematologic AEs, all AEs are graded according to CTCAE version 4.0. Acute GVHD (aGVHD), chronic GVHD (cGVHD) and hematologic AEs are graded as AEs according to our self-defined criteria.

**
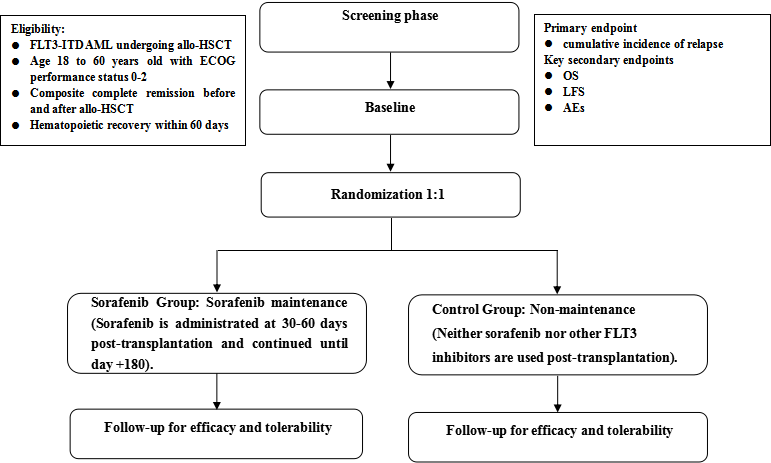
Figure 1 Study Schema**

FLT3-ITD= FLT3 internal tandem duplication; AML= acute myeloid leukemia; allo-HSCT= allogeneic hematopoietic stem cell transplantation; ECOG= Eastern Cooperative Oncology Group; OS= overall survival; LFS= leukemia-free survival; AEs= adverse effects.

# 4. Subject Selection Criteria

## 4.1 Subject Selection Criteria

### 4.1.1 Number of Subjects

Approximately 194 subjects will be randomized to sorafenib or control group (97 subjects per group).

### 4.1.2 Inclusion Criteria

Subjects eligible for enrolment in this study must meet all of the following criteria:

1. Patients with FLT3-ITD AML undergoing first allo-HSCT

2. Age 18 to 60 years old with ECOG performance status 0-2

3. Composite complete remission (CRc) before and after allo-HSCT (CRc must be confirmed by BM analysis within 3 days before randomization).

- CRc comprises complete remission (CR), CR with incomplete platelet recovery (CRp), and CR with incomplete hematologic recovery (CRi).
- CR is defined as one marrow (BM) blasts <5%; absence of circulating blasts and blasts with Auer rods; absence of extramedullary disease; absolute neutrophil count (ANC) ≥1·0×10^9^/L and platelet count (PLT) ≥100×10^9^/L.
- CRp is defined as all CR criteria except for incomplete platelet recovery (<100×10^9^/L).
- CRi is defined as all CR criteria except for incomplete hematologic recovery with residual neutropenia <1·0×10^9^/L and/ or thrombocytopenia <100×10^9^/L.

4. Hematopoietic recovery within 60 days post-transplantation

- Hematopoietic recovery post-transplantation is defined as an ANC ≥ 1.0 × 10^9^/L and a non-transfused PLT ≥ 30 × 10^9^/L within three days.

5. Sign informed consent form, have the ability to comply with study and follow-up procedures

### 4.1.3 Exclusion Criteria

Subjects meeting any of the following criteria are ineligible for this study:

1. Acute promyelocytic leukemia (AML subtype M3)

2. Intolerance to sorafenib pre-transplantation

3. Life expectancy less than 30 days post-transplantation

4. Active aGVHD or uncontrolled infections within 60 days post-transplantation

5. Cardiac dysfunction (particularly congestive heart failure, unstable coronary artery disease and serious cardiac ventricular arrhythmias requiring antiarrhythmic therapy)

6. Respiratory failure (PaO2 ≤60mmHg)

7. Hepatic abnormalities (total bilirubin ≥2 times the upper limit of normal [ULN], alanine aminotransferase [ALT] or aspartate aminotransferase [AST] ≥2 times the ULN)

8. Renal dysfunction (creatinine ≥2 times the ULN or creatinine clearance rate < 30 mL/min)

9. ECOG performance status 3, 4 or 5

10. With any conditions not suitable for the trial (investigators' decision)

## 4.2. Withdrawal Criteria

Subjects are free to withdraw consent and discontinue participation in the study at any time and without prejudice to future treatment. A subject's participation in the study may be discontinued at any time at the investigator's discretion. Justifiable reasons for a subject to be withdrawn from the study include:

1. Inability to fully comply with the study protocol

2. Initiation of alternative treatment of other FLT3 inhibitors post-transplantation

3. Unacceptable toxicity

4. Best interest of the subject based upon the investigator’s discretion

5. At the request of the study subject at any time and for any reason

Subjects will be followed up unless the informed consent is withdrawn. The reason for withdrawal from study participation and the date must be documented in the case report form (CRF). The investigator must complete the last visit, including vital signs, physical examination, laboratory tests, disease status and AE assessment, all of which must be documented in the CRF.

# 5. Study Procedures

## 5.1 Screening

Subjects with FLT3-ITD AML receiving first allo-HSCT will be screened for eligibility. Medical history evaluation, vital sign, physical examination, ECOG performance status, blood and urine sampling for laboratory tests, electrocardiogram, chest imaging examination as well as BM assessment will be performed to determine study eligibility, all of which must be performed ≤3 days prior to randomization.

## 5.2 Treatment Allocation and Blinding

This is an open-label study. Neither subjects nor investigators will be blinded to treatment. Upon completion of all the required screening assessments, eligible subjects will be randomized at 1:1 ratio to receive sorafenib maintenance or non-maintenance post-transplantation. Randomization is done with permuted blocks (block size four), and implemented through an interactive web-based response system.

## 5.3 Study Treatment

## 5.3.1 Sorafenib Group (sorafenib maintenance therapy)

Sorafenib is started from day +30 until day +180 post-transplantation if patients meet the inclusion criterion on day +30. For patients who do not meet the inclusion criterion on day +30 but who do so within 31-60 days post-transplantation, sorafenib is administrated at 31-60 days post-transplantation and continued until day +180. The initial dose of sorafenib is 400 mg orally BID. In cases of grade 3 or worse AEs, dose modifications including dose reductions or interruptions are allowed at the investigator’s discretion. After the resolution of AEs, the dose is re-escalated from 200 mg QD or 200 mg BID to 400 mg BID at the discretion of the investigator.

## 5.3.2 Control Group (non-maintenance therapy)

Neither sorafenib nor other FLT3 inhibitors can be used unless the patients experience relapse.

## 5.4 Follow-up

Disease assessment including routine blood and BM assessment will be regularly performed post-transplantation. BM assessment, including BM smear and FLT3-ITD mutation is performed before randomization, every month for the first three months after enrollment, every two months from the 4th to 9th month after enrollment, and then every three months until the study is completed.

All subjects will be followed for safety and tolerability within 210 days post-transplantation. With the exception of GVHD and hematologic AEs, all AEs are graded according to CTCAE version 4.0. aGVHD, cGVHD and hematologic AEs are graded as AEs according to our self-defined criteria.

# 6. Efficacy Assessments

## 6.1 Definitions

- - Relapse is defined as reappearance of leukemic blasts in the peripheral blood or ≥ 5% blasts in the BM as pirate or biopsy not attributable to any other cause or reappearance or new appearance of extramedullary leukemia.
- CRc comprises CR, CRp, and CRi.
- CR is defined as BM blasts <5%; absence of circulating blasts and blasts with Auer rods; absence of extramedullary disease; ANC ≥1·0×10^9^/L and PLT ≥100×10^9^/L.
- CRp is defined as all CR criteria except for incomplete platelet recovery (<100×10^9^/L).
- CRi is defined as all CR criteria except for incomplete hematologic recovery with residual neutropenia <1·0×10^9^/L and/ or thrombocytopenia <100×10^9^/L.
  - Partial remission (PR) is defined as 5%-20% blasts with or without extramedullary leukemia.
  - No evidence of response (NR) is defined as a failure to obtain PR or better.

## 6.2 Primary Efficacy Endpoint

- The 1-year cumulative incidence of relapse post-transplantation.

## 6.3 Secondary Efficacy Endpoints

- - OS is defined as the time from transplantation until death from any cause.
- LFS is defined as survival in continuous CR without relapse, and refer to the time from transplantation until relapse or death from any cause.

## 6.4 Schedule and methods of Efficacy Assessments

Disease assessment including routine blood and BM assessment will be regularly performed post-transplantation. Routine blood is monitored before randomization, twice a week for the first month after enrollment, once a week from the 2nd to 3rd month after enrollment, and then once every two weeks until the study is completed. BM assessment, including BM smear and FLT3-ITD mutation is performed before randomization, every month for the first three months after enrollment, every two months from the 4th to 9th month after enrollment, and then every three months until the study is completed. Mutation analysis of the FLT3-ITD (exons 14 and 15) gene is performed by polymerase chain reaction and direct sequencing. All subjects who complete treatment or withdraw from the study must receive efficacy assessment.

# 7. Safety Evaluation

All subjects enrolled in the study will be evaluable for safety and tolerability. Safety and tolerability will be assessed with vital signs, physical examination, clinical symptoms, and clinical laboratory evaluations (hematology, serum chemistry, urinalysis, electrocardiogram, and chest imaging examination). With the exception of GVHD and hematologic AEs, all AEs will be evaluated within 210 days post-transplantation according to CTCAE version 4.0. aGVHD, cGVHD and hematologic AEs are graded as AEs according to our self-defined criteria.

### 7.1 Medical History

Each subject's medical history must be obtained at screening. Information on any prior or existing medical conditions will be recorded on the appropriate CRF.

### 7.2 Vital Signs and Physical Examination

Vital signs and results of physical examination must be documented before randomization, once a week for the first month after enrollment, once every two weeks from the 2nd month after enrollment until 210 days post-transplantation. The next 8 items must be performed:

⚫ Physical examination

⚫ Heart rate

⚫ Blood pressure

⚫ Body temperature

⚫ Rate of respiration

⚫ Body weight

⚫ ECOG performance status

⚫ Signs of infection

### 7.3 Clinical Symptoms

During the study, the patients' clinical symptoms must be documented. The clinical symptoms may be associated with the administration of sorafenib reported previously, [^15^](#_ENREF_15) including rash, hand-foot-skin reaction, dermatitis, erythema, flushing, pruritus, dry skin, alopecia, stomatitis, diarrhea, nausea, vomiting, pancreatitis, weight loss, anorexia, fatigue, fever, headache, sensory neuropathy, myalgia, arthralgia, abdominal pain, cardiac ischaemia, dyspnea, infections, bleeding and hypertension. Besides, some retrospective studies have demonstrated that application of sorafenib post-transplantation might increase the incidence of GVHD.[^16^](#_ENREF_16)^,^ [^17^](#_ENREF_17)

### 7.4 Clinical Laboratory Evaluations

Before initiation of the study, the monitors will document the normal range of each test in every involved laboratory. During the study, the next items must be performed:

⚫ Routine blood: white cell counts, neutrophil cell counts, hemoglobin, and platelet counts

⚫ Hepatic function: total bilirubin (both direct bilirubin and indirect bilirubin must be documented when the total bilirubin elevates), ALT, AST, lactic dehydrogenase, alkaline phosphatase, albumin and total protein

- Renal function: serum creatinine, urea nitrogen and uric acid
- Other biochemical indicators: amylase and lipase
- Electrolytes: sodium, potassium, calcium and magnesium
- Coagulation function: prothrombin time, prothrombin time-international normalized ratio, activated partial thromboplastin time and fibrinogen
- Urinalysis: protein, glucose and erythrocyte
- Electrocardiogram
- Chest imaging examination

## 8 Adverse Events and Serious Adverse Events (SAEs)

The investigator is responsible for detecting, documenting and reporting events that meet the definition of an AE or SAE.

### 8.1. Definitions

## 8.1.1 Adverse Events

An AE is any untoward medical occurrence in a subject of a clinical investigation, which does not necessarily have a causal relationship to the medicinal product. Therefore, an AE can be any unfavorable and unintended sign, including an abnormal laboratory finding, symptom, or disease (new or exacerbated), whether or not it is considered to be related to the product. This definition includes any newly occurring event or previous condition that has increased in severity or frequency since the administration of the product. However, relapse or death due to relapse should not be recorded as AEs.

## 8.1.2 Serious Adverse Events

A serious adverse event is any untoward medical occurrence that, at any dose:

- - Results in death
  - Is life-threatening
  - Requires hospitalization or prolongation of existing hospitalization - ie, the AE requires at least a 24-hour inpatient hospitalization or prolongs a hospitalization beyond the expected length of stay.

Hospitalization or prolongation of existing hospitalization for social reasons will not be reported as an SAE.

- - Results in disability/incapacity
  - Congenital anomaly/ birth defect
  - Important medical event

Medical or scientific judgment should be exercised in deciding whether SAE reporting is appropriate in other situations. An important medical event is an event that may not result in death, be life-threatening, or require hospitalization, but is clearly of major clinical significance. The AE may jeopardize the subject or require intervention to prevent a serious outcome.

## 8.2 Assessment of Severity

With the exception of GVHD and hematologic AEs, all AEs are graded according to CTCAE version 4.0. Considering our inclusion criteria are ANC ≥ 1.0×10^9^/L and PLT ≥ 30×10^9^/L within 60 days post-transplantation, grade 3 hematologic AEs are defined as ANC < 1.0-0.5×10^9^/L or PLT < 30-20×10^9^/L, and grade 4 hematologic AEs as ANC < 0.5×10^9^/L or PLT < 20×10^9^/L. When CTCAE version 4.0 criteria do not apply, severity will be defined according to the following criteria:

| **Severity** | **Description** |
| --- | --- |
| Grade 1- Mild | Asymptomatic or mild symptoms; clinical or diagnostic observations only; intervention not indicated |
| Grade 2- Moderate | Minimal, local or noninvasive intervention indicated; limiting age-appropriate instrumental activities of daily living (ADL) |
| Grade 3- Severe | Medically significant but not immediately life threatening; hospitalization or prolongation of hospitalization indicated; disabling; limiting self-case ADL |
| Grade 4- Life-threatening | Life-threatening consequences; urgent intervention indicated |
| Grade 5- Death | Death |

In this study, aGVHD is graded according to the 1994 Consensus Conference on Acute GVHD Grading,[^18^](#_ENREF_18) and cGVHD is graded according to the National Institutes of Health (NIH) criteria.[^19^](#_ENREF_19) aGVHD and cGVHD are graded as AEs according to the above criteria. Grade I aGVHD without intervention is graded as grade 1 AE, grade I aGVHD with intervention as grade 2 AE, grade II aGVHD as grade 3 AE, grade III-IV aGVHD as grade 4 AE, and death due to aGVHD as grade 5 AE. With regards to cGVHD, mild cGVHD without systematic intervention is graded as grade 1 AE, mild cGVHD with systematic intervention as grade 2 AE, moderate cGVHD as grade 3 AE, severe cGVHD as grade 4 AE, and death due to cGVHD as grade 5 AE.

## 8.3 Assessment of Causality

The investigator must determine the relationship of each AE and SAE to study treatment. Relationship of an AE or SAE to study treatment will be defined according to the following criteria:

- - Definite: There is a clear temporal relationship to study treatment, with no other possible cause.
  - Possible: A temporal relationship to study treatment is not clear, and alternative etiologies are possible.
  - Not related: There is no temporal relationship to study treatment, and/or there is evidence of an alternative cause such as a concurrent medication or illness.

## 8.4 Recording and Reporting AEs and SAEs

All AEs and SAEs must be recorded in the appropriate CRF, whether or not they are associated to be causally related to study treatment. Each SAE must be reported promptly on the Serious Adverse Event Report Form, and submitted to the Independent Ethics Committee within 24 hours by the investigator. The information recorded on the Serious Adverse Event Report Form will include at least the following: subject number, identity of the event, study drug name and dose, investigator's assessment of the event's severity and relationship to study treatment, and investigator's name and signature. Clinical monitors must collect and verify detailed information of AEs and SAEs when examining original medical records. All AEs and SAEs should be followed up until resolved.

# 9. Rules of Withdrawal

## 9.1. Subjects Withdraw from the Study

Subjects can withdraw from the study at any time for any reason without impact on the investigator’s right to treat disease for subjects. Based upon the interest of subjects, the investigator has the right to request subjects to withdraw from the study for any reason including concomitant disease, AEs or treatment failure. The core group of clinical study reserves the right to request subjects to withdraw from the study for deviation(s) from the protocol, administrative reasons, or other effective or ethical reasons.

The last assessment for subjects must be performed and documented in the CRF regardless of the time and reason for withdrawal. The reason for withdrawal from study participation must be documented in the CRF. All documents related to subjects should be completed. Despite withdrawal from the study, those subjects should be followed up and documented about their diseases until withdrawal of informed consents.

For subjects who withdraw from the study due to concomitant diseases or AEs, the details must be documented in the CRF with other appropriate and valuable data attached.

## 9.2. Premature Termination of the Study

Reasons for premature termination of the study include external events, repetition of SAEs, growing incidence of treatment-related death and slow enrolment in the study. All subjects will be informed of premature termination of the study by written consents. Any subjects who decide to discontinue participating in the study must report to the principal investigator.

# 10. Rules of Follow-Up

## 10.1 Follow-up Period

Starting from randomization.

## 10.2 Visit Scheduling

Every week for the first three months after enrollment, and then every month until the study is completed.

## 10. 3 Contents

The contents of every follow-up visit include complaints of subjects, vital signs, physical examination, clinical symptoms and clinical laboratory evaluations (hematology, serum chemistry, urinalysis, electrocardiogram, chest imaging examination, and BM assessment). All of the results must be documented in the original medical record.

# 11. Data Analysis and Statistical Considerations

## 11.1. Hypotheses

The primary endpoint is the 1-year cumulative incidence of relapse post-transplantation. The null and alternative hypotheses are designed with the goal of demonstrating the superiority of sorafenib maintenance over non-maintenance post-transplantation with respect to relapse. Superiority will be determined using the following hypothesis:

H0: incidence of relapse with sorafenib maintenance ≥ incidence of relapse with non-maintenance post-transplantation

H1: incidence of relapse with sorafenib maintenance < incidence of relapse with non-maintenance post-transplantation

## 11.2 Study Design Considerations

This prospective, open-label, multicenter, randomized, phase 3 study compares the efficacy of sorafenib maintenance and non-maintenance post-transplantation in FLT3-ITD AML undergoing allo-HSCT. The primary outcome is the 1-year cumulative incidence of relapse post-transplantation, and the study is designed to determine if sorafenib maintenance is superior to non-maintenance post-transplantation in the study population with respect to relapse. Base on the reported 1-year cumulative incidence of relapse of 40% for non-maintenance post-transplantation in FLT3-ITD AML undergoing allo-HSCT,[^20^](#_ENREF_20) a clinically meaningful reduction in 1-year cumulative incidence of relapse would be 20% for sorafenib maintenance post-transplantation.

### 11.2.1 Sample Size Assumptions

The sample size calculation is based on the primary endpoint, the 1-year cumulative incidence of relapse post-transplantation, with the following assumptions:

- - - - the 1-year cumulative incidence of relapse in the control group: 40%
      - the 1-year cumulative incidence of relapse in the sorafenib group: 20%
      - a 1:1 randomization scheme
      - a 5% one-tailed risk of erroneously claiming a difference in the presence of no true underlying difference by z-test with pooled variance
      - a 90% chance of successfully declaring a difference in the presence of a true underlying difference (power)
      - 10% percent of cases drop

Under the above assumptions, a total sample size of 194 subjects is required (97 in each group).

### 11.2.2 Primary Efficacy Endpoint

The primary efficacy endpoint is the 1-year cumulative incidence of relapse post-transplantation.

### 11.2.3 Secondary Efficacy Endpoints

The secondary efficacy endpoints include OS and LFS.

## 11.3 Data Analysis Considerations

### 11.3.1 Analysis Population

The primary population will be the intent-to-treat (ITT) population, which is defined as all subjects randomized to the two groups. This ITT population will be the basis for the analysis of efficacy and safety endpoints in this study.

### 11.3.2 Analysis Plan

### 11.3.2.1 Baseline Data

Baseline characteristics will be summarized and described in a frequency list.

### 11.3.2.2 Analysis of Efficacy

The definition of efficacy endpoints has been detailed in previous section. Cumulative incidence of relapse post-transplantation is calculated by accounting for competing risk. Non-relapse mortality is considered a competing risk for relapse. The comparison of the cumulative incidence of relapse is tested using Fine and Gray model. OS and LFS are estimated using the Kaplan-Meier method and compared using the log-rank test. The corresponding HR and 95% CI were estimated using the Cox proportional hazards model. All statistical tests are two-tailed with a significance level of 0.05. SPSS 20.0 (SPSS Inc., Chicago, IL, USA) and R version 3.3.0 (R Development Core Team, Vienna, Austria) are used for all data analysis.

### 11.3.2.3 Analysis of Safety

Safety and tolerability will be assessed by incidence and severity of AEs and changes from baseline of all relevant parameters, including laboratory test values, physical examination, vital signs, and ECOG performance scores. The definition of AEs has been detailed in previous section. With the exception of GVHD and hematologic AEs, all AEs are graded according to CTCAE version 4.0. aGVHD, cGVHD, and hematologic AEs are graded as AEs according to our self-defined criteria. All subjects will be monitored for AEs within 210 days post-transplantation. Categorical data will be summarized by proportion of total subjects. Quantitative data will be described using arithmetic average or median for central tendency and standard deviation or interquartile range for distribution range.

# 12. Materials for the Study

All materials provided to study sites and investigators are as follows:

- The study protocol
- Informed consent
- CRF

# 13. Ethical Considerations

## 13.1 Responsibility of Investigators

The investigators have the responsibility for guarantee of the clinical study’s compliance with the protocol, Chinese good clinical practice (GCP) guidelines and applicable laws and regulations.

## 13.2 Informed Consent Process

Prior to participation in the study, subjects must be informed about objectives, methods, possible benefits, potential risks and possible discomforts of the study by investigators. They also should be informed that participation in the study would be voluntary, they can withdraw from the study at any time, there is no impact on the treatment of the disease whether they take part in the study and their privacy will be protected.

Subjects or their legally acceptable representative should have enough time to read the inform consent and raise queries. Written informed consent must be obtained from each subject, or their legally acceptable representative.

## 13.3 Good Clinical Practice

This study will be conducted in accordance with the Declaration of Helsinki and Chinese GCP. The study will be conducted only if it is approved by the ethical review committee of the principal study site. The investigators will guarantee that the study will be conducted in accordance with applicable laws and regulations, scientific and ethical principles of the People’s Republic of China. If the protocol needs revision during the study, the revised version must be reapproved by the ethical review committee of the principal study site. If new data related to study treatment are discovered, the informed consent must be revised and the revision must be reapproved by the ethical review committee of the principal study site and subjects.

## 13.4 Protection of Subjects’ Personal Data

Data collected in the study are limited to the efficacy and safety related to study treatment. Data will be collected and used in accordance with applicable laws and regulations.

# 14. Administrative Requirements

Neither the investigator nor the applicant can revise the protocol without agreement of the opposite side. All revisions of the protocol must be released by the applicant institution. To insure the integrity, accuracy and reliability of the data, relevant results of examination and treatment must be documented in original medical record and CRF. Independent clinical monitoring is performed regularly by a panel of qualified and experienced study investigators composed of hematologists who are blinded as to the treatment assignments.

# 15. References

1. Shannon K, Armstrong SA. Genetics, epigenetics, and leukemia. *N Engl J Med* 2010; 363: 2460-1.

2. Gilliland DG, Griffin JD. The roles of FLT3 in hematopoiesis and leukemia. *Blood* 2002; 100: 1532-42.

3. Thiede C, Steudel C, Mohr B, et al. Analysis of FLT3-activating mutations in 979 patients with acute myelogenous leukemia: association with FAB subtypes and identification of subgroups with poor prognosis. *Blood* 2002; 99: 4326-35.

4. Frohling S, Schlenk RF, Breitruck J, et al. Prognostic significance of activating FLT3 mutations in younger adults (16 to 60 years) with acute myeloid leukemia and normal cytogenetics: a study of the AML Study Group Ulm. *Blood* 2002; 100: 4372-80.

5. Sengsayadeth SM, Jagasia M, Engelhardt BG, et al. Allo-SCT for high-risk AML-CR1 in the molecular era: impact of FLT3/ITD outweighs the conventional markers. *Bone Marrow Transplant* 2012; 47: 1535-7.

6. DeZern AE, Sung A, Kim S, et al. Role of allogeneic transplantation for FLT3/ITD acute myeloid leukemia: outcomes from 133 consecutive newly diagnosed patients from a single institution. *Biol Blood Marrow Transplant* 2011; 17: 1404-9.

7. Wilhelm S, Carter C, Lynch M, et al. Discovery and development of sorafenib: a multikinase inhibitor for treating cancer. *Nat Rev Drug Discov* 2006; 5: 835-44.

8. Zhang W, Konopleva M, Shi YX, et al. Mutant FLT3: a direct target of sorafenib in acute myelogenous leukemia. *J Natl Cancer Inst* 2008; 100: 184-98.

9. Ravandi F, Cortes JE, Jones D, et al. Phase I/II study of combination therapy with sorafenib, idarubicin, and cytarabine in younger patients with acute myeloid leukemia. *J Clin Oncol* 2010; 28: 1856-62.

10. Ravandi F, Arana Yi C, Cortes JE, et al. Final report of phase II study of sorafenib, cytarabine and idarubicin for initial therapy in younger patients with acute myeloid leukemia. *Leukemia* 2014; 28: 1543-5.

11. Ravandi F, Alattar ML, Grunwald MR, et al. Phase 2 study of azacytidine plus sorafenib in patients with acute myeloid leukemia and FLT-3 internal tandem duplication mutation. *Blood* 2013; 121: 4655-62.

12. Chen YB, Li S, Lane AA, et al. Phase I trial of maintenance sorafenib after allogeneic hematopoietic stem cell transplantation for fms-like tyrosine kinase 3 internal tandem duplication acute myeloid leukemia. *Biol Blood Marrow Transplant* 2014; 20: 2042-8.

13. Antar A, Kharfan-Dabaja MA, Mahfouz R, Bazarbachi A. Sorafenib Maintenance Appears Safe and Improves Clinical Outcomes in FLT3-ITD Acute Myeloid Leukemia After Allogeneic Hematopoietic Cell Transplantation. *Clin Lymphoma Myeloma Leuk* 2015; 15: 298-302.

14. Sammons SL, Pratz KW, Smith BD, Karp JE, Emadi A. Sorafenib is tolerable and improves clinical outcomes in patients with FLT3-ITD acute myeloid leukemia prior to stem cell transplant and after relapse post-transplant. *Am J Hematol* 2014; 89: 936-8.

15. Takimoto CH, Awada A. Safety and anti-tumor activity of sorafenib (Nexavar) in combination with other anti-cancer agents: a review of clinical trials. *Cancer Chemother Pharmacol* 2008; 61: 535-48.

16. Yokoyama H, Lundqvist A, Su S, Childs R. Toxic effects of sorafenib when given early after allogeneic hematopoietic stem cell transplantation. *Blood* 2010; 116: 2858-9.

17. Metzelder S, Wang Y, Wollmer E, et al. Compassionate use of sorafenib in FLT3-ITD-positive acute myeloid leukemia: sustained regression before and after allogeneic stem cell transplantation. *Blood* 2009; 113: 6567-71.

18. Przepiorka D, Weisdorf D, Martin P, et al. 1994 Consensus Conference on Acute GVHD Grading. *Bone Marrow Transplant* 1995; 15: 825-8.

19. Jagasia MH, Greinix HT, Arora M, et al. National Institutes of Health Consensus Development Project on Criteria for Clinical Trials in Chronic Graft-versus-Host Disease: I. The 2014 Diagnosis and Staging Working Group report. *Biol Blood Marrow Transplant* 2015; 21: 389-401 e1.

20. Brunet S, Martino R, Sierra J. Hematopoietic transplantation for acute myeloid leukemia with internal tandem duplication of FLT3 gene (FLT3/ITD). *Curr Opin Oncol* 2013; 25: 195-204.

# 16 Appendices

## 16.1 Appendix 1 Diagnosis and Classification of aGVHD and cGVHD

**Grading of aGVHD**

| Grade | Degree of organ involvement |
| --- | --- |
| I | Stage 1-2 skin rash; no gut involvement; no liver involvement; no decrease in clinical performance |
| II | Stage 1-3 skin rash; stage 1 gut involvement or stage 1 liver involvement (or both); mild decrease in clinical performance |
| III | Stage 2-3 skin rash; stage 2-3 gut involvement or 2-4 liver involvement (or both); marked decrease in clinical performance |
| IV | Similar to Grade III with stage 2-4 organ involvement and extreme decrease in clinical performance |

Przepiorka D, Weisdorf D, Martin P, [Klingemann HG](https://www.ncbi.nlm.nih.gov/pubmed/?term=Klingemann%20HG%5BAuthor%5D&cauthor=true&cauthor_uid=7581076), [Beatty P](https://www.ncbi.nlm.nih.gov/pubmed/?term=Beatty%20P%5BAuthor%5D&cauthor=true&cauthor_uid=7581076), [Hows J](https://www.ncbi.nlm.nih.gov/pubmed/?term=Hows%20J%5BAuthor%5D&cauthor=true&cauthor_uid=7581076), [Thomas ED](https://www.ncbi.nlm.nih.gov/pubmed/?term=Thomas%20ED%5BAuthor%5D&cauthor=true&cauthor_uid=7581076). 1994 Consensus Conference on Acute GVHD Grading. Bone Marrow Transplant. 1995; 15(6):825-828.

**Grading of cGVHD**

| NIH Global Severity of chronic GVHD |
| --- |
| Mild chronic GVHD |
| 1 or 2 Organs involved with no more than score 1 plus Lung score 0 |
| Moderate chronic GVHD |
| 3 or More organs involved with no more than score 1 |
| OR |
| At least 1 organ (not lung) with a score of 2 |
| OR |
| Lung score 1 |
| Severe chronic GVHD |
| At least 1 organ with a score of 3 |
| OR |
| Lung score of 2 or 3 |
| Key points:  In skin: higher of the 2 scores to be used for calculating global severity.  In lung: FEV1 is used instead of clinical score for calculating global severity.  If the entire abnormality in an organ is noted to be unequivocally explained by a non-GVHD documented cause, that organ is not included for calculation of the global severity.  If the abnormality in an organ is attributed to multifactorial causes (GVHD plus other causes), the scored organ will be used for calculation of the global severity regardless of the contributing causes (no downgrading of organ severity score). |

Jagasia MH, Greinix HT, Arora M,  [Williams KM](https://www.ncbi.nlm.nih.gov/pubmed/?term=Williams%20KM%5BAuthor%5D&cauthor=true&cauthor_uid=25529383), [Wolff D](https://www.ncbi.nlm.nih.gov/pubmed/?term=Wolff%20D%5BAuthor%5D&cauthor=true&cauthor_uid=25529383), [Cowen EW](https://www.ncbi.nlm.nih.gov/pubmed/?term=Cowen%20EW%5BAuthor%5D&cauthor=true&cauthor_uid=25529383), [Palmer J](https://www.ncbi.nlm.nih.gov/pubmed/?term=Palmer%20J%5BAuthor%5D&cauthor=true&cauthor_uid=25529383), [Weisdorf D](https://www.ncbi.nlm.nih.gov/pubmed/?term=Weisdorf%20D%5BAuthor%5D&cauthor=true&cauthor_uid=25529383), [Treister NS](https://www.ncbi.nlm.nih.gov/pubmed/?term=Treister%20NS%5BAuthor%5D&cauthor=true&cauthor_uid=25529383), [Cheng GS](https://www.ncbi.nlm.nih.gov/pubmed/?term=Cheng%20GS%5BAuthor%5D&cauthor=true&cauthor_uid=25529383), [Kerr H](https://www.ncbi.nlm.nih.gov/pubmed/?term=Kerr%20H%5BAuthor%5D&cauthor=true&cauthor_uid=25529383), [Stratton P](https://www.ncbi.nlm.nih.gov/pubmed/?term=Stratton%20P%5BAuthor%5D&cauthor=true&cauthor_uid=25529383), [Duarte RF](https://www.ncbi.nlm.nih.gov/pubmed/?term=Duarte%20RF%5BAuthor%5D&cauthor=true&cauthor_uid=25529383), [McDonald GB](https://www.ncbi.nlm.nih.gov/pubmed/?term=McDonald%20GB%5BAuthor%5D&cauthor=true&cauthor_uid=25529383), [Inamoto Y](https://www.ncbi.nlm.nih.gov/pubmed/?term=Inamoto%20Y%5BAuthor%5D&cauthor=true&cauthor_uid=25529383), [Vigorito A](https://www.ncbi.nlm.nih.gov/pubmed/?term=Vigorito%20A%5BAuthor%5D&cauthor=true&cauthor_uid=25529383), [Arai S](https://www.ncbi.nlm.nih.gov/pubmed/?term=Arai%20S%5BAuthor%5D&cauthor=true&cauthor_uid=25529383), [Datiles MB](https://www.ncbi.nlm.nih.gov/pubmed/?term=Datiles%20MB%5BAuthor%5D&cauthor=true&cauthor_uid=25529383), [Jacobsohn D](https://www.ncbi.nlm.nih.gov/pubmed/?term=Jacobsohn%20D%5BAuthor%5D&cauthor=true&cauthor_uid=25529383), [Heller T](https://www.ncbi.nlm.nih.gov/pubmed/?term=Heller%20T%5BAuthor%5D&cauthor=true&cauthor_uid=25529383), [Kitko CL](https://www.ncbi.nlm.nih.gov/pubmed/?term=Kitko%20CL%5BAuthor%5D&cauthor=true&cauthor_uid=25529383), [Mitchell SA](https://www.ncbi.nlm.nih.gov/pubmed/?term=Mitchell%20SA%5BAuthor%5D&cauthor=true&cauthor_uid=25529383), [Martin PJ](https://www.ncbi.nlm.nih.gov/pubmed/?term=Martin%20PJ%5BAuthor%5D&cauthor=true&cauthor_uid=25529383), [Shulman H](https://www.ncbi.nlm.nih.gov/pubmed/?term=Shulman%20H%5BAuthor%5D&cauthor=true&cauthor_uid=25529383), [Wu RS](https://www.ncbi.nlm.nih.gov/pubmed/?term=Wu%20RS%5BAuthor%5D&cauthor=true&cauthor_uid=25529383), [Cutler CS](https://www.ncbi.nlm.nih.gov/pubmed/?term=Cutler%20CS%5BAuthor%5D&cauthor=true&cauthor_uid=25529383), [Vogelsang GB](https://www.ncbi.nlm.nih.gov/pubmed/?term=Vogelsang%20GB%5BAuthor%5D&cauthor=true&cauthor_uid=25529383), [Lee SJ](https://www.ncbi.nlm.nih.gov/pubmed/?term=Lee%20SJ%5BAuthor%5D&cauthor=true&cauthor_uid=25529383), [Pavletic SZ](https://www.ncbi.nlm.nih.gov/pubmed/?term=Pavletic%20SZ%5BAuthor%5D&cauthor=true&cauthor_uid=25529383), [Flowers ME](https://www.ncbi.nlm.nih.gov/pubmed/?term=Flowers%20ME%5BAuthor%5D&cauthor=true&cauthor_uid=25529383). National Institutes of Health Consensus Development Project on Criteria for Clinical Trials in Chronic Graft-versus-Host Disease: I. The 2014 Diagnosis and Staging Working Group report. Biol Blood Marrow Transplant. 2015; 21(3): 389-401.e1.


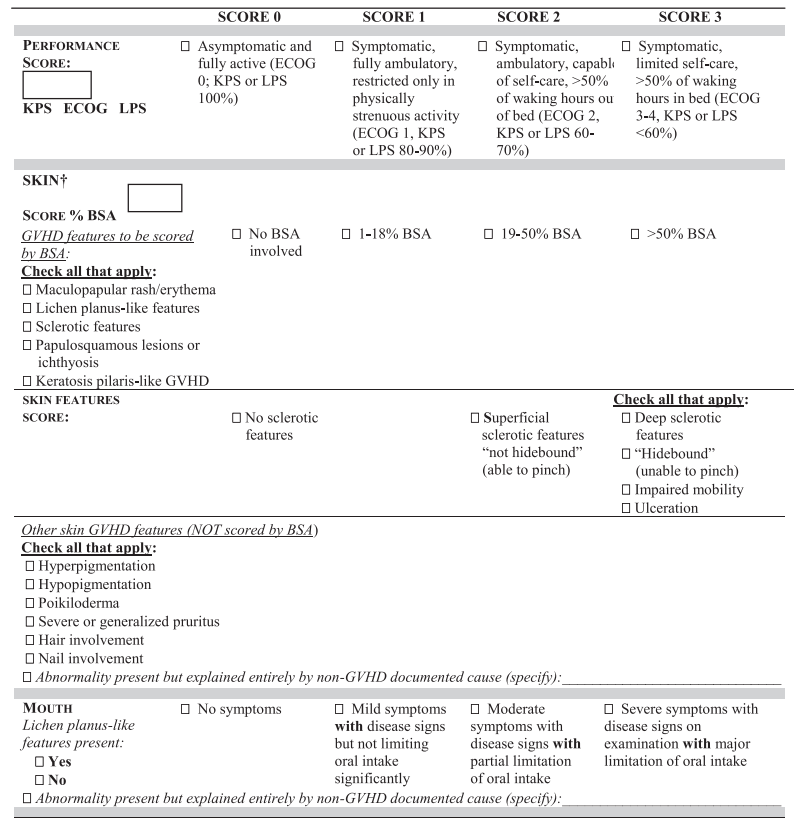


Figure 1. Organ scoring of chronic GVHD. ECOG indicates Eastern Cooperative Oncology Group; KPS, Karnofsky Performance Status; LPS, Lansky Performance Status; BSA, body surface area; ADL, activities of daily living; LFTs, liver function tests; AP, alkaline phosphatase; ALT, alanine aminotransferase; ULN, normal upper limit. *Weight loss within 3 months. †Skin scoring should use both percentage of BSA involved by disease signs and the cutaneous features scales. When a discrepancy exists between the percentage of total body surface (BSA) score and the skin feature score, OR if superfificial sclerotic features are present (Score 2), but there is impaired mobility or ulceration (Score 3), the higher level should be used for the final skin scoring. **Lung scoring should be performed using both the symptoms and FEV1 scores whenever possible. FEV1 should be used in the final lung scoring where there is discrepancy between symptoms and FEV1 scores.


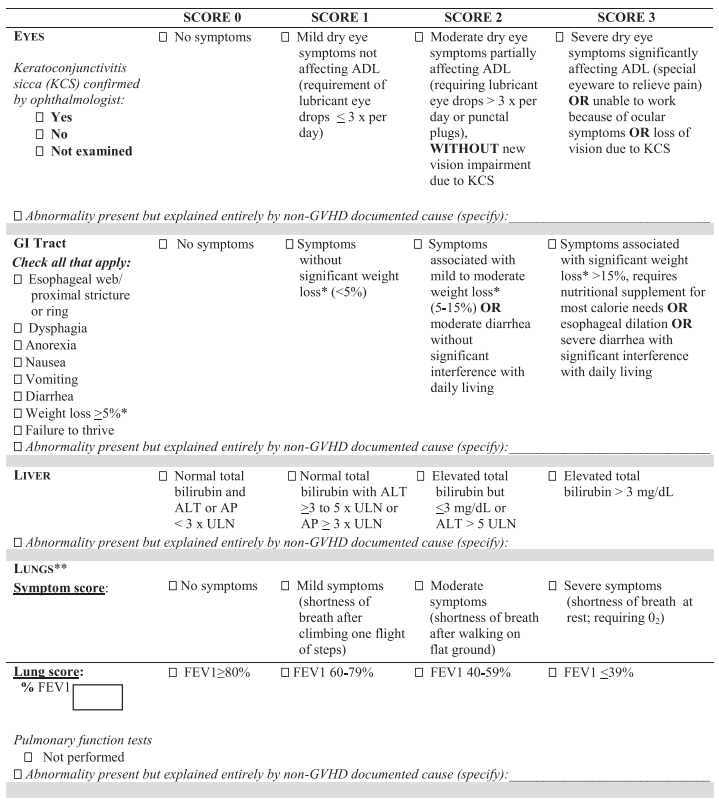


Figure 1. (continued).


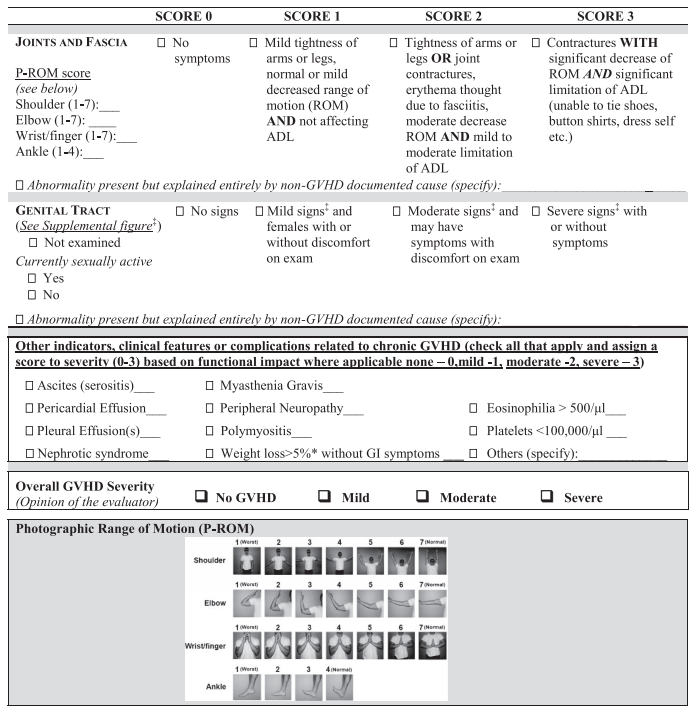


Figure 1. (continued).

Jagasia MH, Greinix HT, Arora M,  [Williams KM](https://www.ncbi.nlm.nih.gov/pubmed/?term=Williams%20KM%5BAuthor%5D&cauthor=true&cauthor_uid=25529383), [Wolff D](https://www.ncbi.nlm.nih.gov/pubmed/?term=Wolff%20D%5BAuthor%5D&cauthor=true&cauthor_uid=25529383), [Cowen EW](https://www.ncbi.nlm.nih.gov/pubmed/?term=Cowen%20EW%5BAuthor%5D&cauthor=true&cauthor_uid=25529383), [Palmer J](https://www.ncbi.nlm.nih.gov/pubmed/?term=Palmer%20J%5BAuthor%5D&cauthor=true&cauthor_uid=25529383), [Weisdorf D](https://www.ncbi.nlm.nih.gov/pubmed/?term=Weisdorf%20D%5BAuthor%5D&cauthor=true&cauthor_uid=25529383), [Treister NS](https://www.ncbi.nlm.nih.gov/pubmed/?term=Treister%20NS%5BAuthor%5D&cauthor=true&cauthor_uid=25529383), [Cheng GS](https://www.ncbi.nlm.nih.gov/pubmed/?term=Cheng%20GS%5BAuthor%5D&cauthor=true&cauthor_uid=25529383), [Kerr H](https://www.ncbi.nlm.nih.gov/pubmed/?term=Kerr%20H%5BAuthor%5D&cauthor=true&cauthor_uid=25529383), [Stratton P](https://www.ncbi.nlm.nih.gov/pubmed/?term=Stratton%20P%5BAuthor%5D&cauthor=true&cauthor_uid=25529383), [Duarte RF](https://www.ncbi.nlm.nih.gov/pubmed/?term=Duarte%20RF%5BAuthor%5D&cauthor=true&cauthor_uid=25529383), [McDonald GB](https://www.ncbi.nlm.nih.gov/pubmed/?term=McDonald%20GB%5BAuthor%5D&cauthor=true&cauthor_uid=25529383), [Inamoto Y](https://www.ncbi.nlm.nih.gov/pubmed/?term=Inamoto%20Y%5BAuthor%5D&cauthor=true&cauthor_uid=25529383), [Vigorito A](https://www.ncbi.nlm.nih.gov/pubmed/?term=Vigorito%20A%5BAuthor%5D&cauthor=true&cauthor_uid=25529383), [Arai S](https://www.ncbi.nlm.nih.gov/pubmed/?term=Arai%20S%5BAuthor%5D&cauthor=true&cauthor_uid=25529383), [Datiles MB](https://www.ncbi.nlm.nih.gov/pubmed/?term=Datiles%20MB%5BAuthor%5D&cauthor=true&cauthor_uid=25529383), [Jacobsohn D](https://www.ncbi.nlm.nih.gov/pubmed/?term=Jacobsohn%20D%5BAuthor%5D&cauthor=true&cauthor_uid=25529383), [Heller T](https://www.ncbi.nlm.nih.gov/pubmed/?term=Heller%20T%5BAuthor%5D&cauthor=true&cauthor_uid=25529383), [Kitko CL](https://www.ncbi.nlm.nih.gov/pubmed/?term=Kitko%20CL%5BAuthor%5D&cauthor=true&cauthor_uid=25529383), [Mitchell SA](https://www.ncbi.nlm.nih.gov/pubmed/?term=Mitchell%20SA%5BAuthor%5D&cauthor=true&cauthor_uid=25529383), [Martin PJ](https://www.ncbi.nlm.nih.gov/pubmed/?term=Martin%20PJ%5BAuthor%5D&cauthor=true&cauthor_uid=25529383), [Shulman H](https://www.ncbi.nlm.nih.gov/pubmed/?term=Shulman%20H%5BAuthor%5D&cauthor=true&cauthor_uid=25529383), [Wu RS](https://www.ncbi.nlm.nih.gov/pubmed/?term=Wu%20RS%5BAuthor%5D&cauthor=true&cauthor_uid=25529383), [Cutler CS](https://www.ncbi.nlm.nih.gov/pubmed/?term=Cutler%20CS%5BAuthor%5D&cauthor=true&cauthor_uid=25529383), [Vogelsang GB](https://www.ncbi.nlm.nih.gov/pubmed/?term=Vogelsang%20GB%5BAuthor%5D&cauthor=true&cauthor_uid=25529383), [Lee SJ](https://www.ncbi.nlm.nih.gov/pubmed/?term=Lee%20SJ%5BAuthor%5D&cauthor=true&cauthor_uid=25529383), [Pavletic SZ](https://www.ncbi.nlm.nih.gov/pubmed/?term=Pavletic%20SZ%5BAuthor%5D&cauthor=true&cauthor_uid=25529383), [Flowers ME](https://www.ncbi.nlm.nih.gov/pubmed/?term=Flowers%20ME%5BAuthor%5D&cauthor=true&cauthor_uid=25529383). National Institutes of Health Consensus Development Project on Criteria for Clinical Trials in Chronic Graft-versus-Host Disease: I. The 2014 Diagnosis and Staging Working Group report. Biol Blood Marrow Transplant. 2015; 21(3): 389-401.e1.

## 16.2 Appendix 2

**ECOG Performance Status**

| **Grade** | **ECOG** |
| --- | --- |
| **0** | Fully active, able to carry on all pre-disease performance without restriction |
| **1** | Restricted in physically strenuous activity but ambulatory and able to carry out work of a light or sedentary nature, e.g., light house work, office work |
| **2** | Ambulatory and capable of all selfcare but unable to carry out any work activities. Up and about more than 50% of waking hours |
| **3** | Capable of only limited selfcare, confined to bed or chair more than 50% of waking hours |
| **4** | Completely disabled. Cannot carry on any selfcare. Totally confined to bed or chair |
| **5** | Dead |

Oken MM, Creech RH, Tormey DC, Horton J, Davis TE, McFadden ET, Carbone PP. Toxicity And Response Criteria Of The Eastern Cooperative Oncology Group. Am J Clin Oncol. 1982; 5(6):649-655.
